# New Bacterial Aryl Sulfotransferases: Effective Tools for Sulfation of Polyphenols

**DOI:** 10.1021/acs.jafc.4c06771

**Published:** 2024-10-01

**Authors:** Katerina Brodsky, Barbora Petránková, Lucie Petrásková, Helena Pelantová, Vladimír Křen, Kateřina Valentová, Pavla Bojarová

**Affiliations:** † Institute of Microbiology of the Czech Academy of Sciences, Vídeňská 1083, Prague 4 CZ-142 00, Czech Republic; ‡ Department of Biochemistry and Microbiology, University of Chemistry and Technology Prague, Technická 3, Prague 6 CZ-166 28, Czech Republic; § Department of Genetics and Microbiology, Faculty of Science, Charles University, Albertov 6, Prague 2 CZ-128 43, Czech Republic

**Keywords:** aryl sulfotransferase, enzymatic sulfation, kaempferol sulfate, metabolite, polyphenol

## Abstract

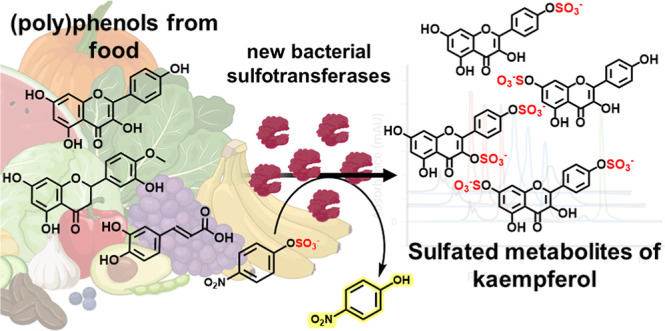

The preparation of pure metabolites of bioactive compounds, particularly (poly)phenols, is essential for the accurate determination of their pharmacological profiles *in vivo*. Since the extraction of these metabolites from biological material is tedious and impractical, they can be synthesized enzymatically *in vitro* by bacterial PAPS-independent aryl sulfotransferases (ASTs). However, only a few ASTs have been studied and used for (poly)phenol sulfation. This study introduces new fully characterized recombinant ASTs selected according to their similarity to the previously characterized ASTs. These enzymes, produced in *Escherichia coli*, were purified, biochemically characterized, and screened for the sulfation of nine flavonoids and two phenolic acids using *p-*nitrophenyl sulfate. All tested compounds were proved to be substrates for the new ASTs, with kaempferol and luteolin being the best converted acceptors. ASTs from *Desulfofalx alkaliphile* (*Dal*AST) and *Campylobacter fetus* (*Cf*AST) showed the highest efficiency in the sulfation of tested polyphenols. To demonstrate the efficiency of the present sulfation approach, a series of new authentic metabolite standards, regioisomers of kaempferol sulfate, were enzymatically produced, isolated, and structurally characterized.

## Introduction

(Poly)phenols, including flavonoids, are a large group of secondary metabolites in higher plants, which are widely distributed in vegetables, fruits, and seeds.^1^ These potent bioactive compounds act as free radical scavengers and anti-inflammatory agents that interact with various endogenous proteins.^2,3^ Despite their importance in human nutrition, (poly)phenols pose a challenge for effective tracking in the organism due to their rapid metabolism. They are preferentially sulfated, methylated, or glucuronylated in phase II biotransformation, and the conjugates often remain in the bloodstream longer than the parent compounds.

Sulfation, *i.e*., the attachment of a sulfate group to an acceptor compound, plays a crucial role in modulating the pharmacokinetic and pharmacodynamic properties of flavonoids. The incorporation of sulfate can significantly improve the water solubility, bioavailability, and stability of these natural compounds, thereby affecting their absorption and distribution in the human body.^4,5^ In addition, sulfation can have an impact on various biological activities.^6−9^ The increasing interest in synthesizing sulfated flavonoid metabolites stems from the realization that sulfated derivatives may exhibit enhanced bioactivity, making them promising candidates for drug development and therapeutic intervention.^10,11^ With the authentic sulfated metabolites in hand, researchers can establish reference points for analytical techniques that enable accurate tracking of biotransformation processes and elucidation of their pharmacokinetics, contributing to advances in nutritional and medicinal analytics and targeted therapeutic interventions.

**Figure 1:**
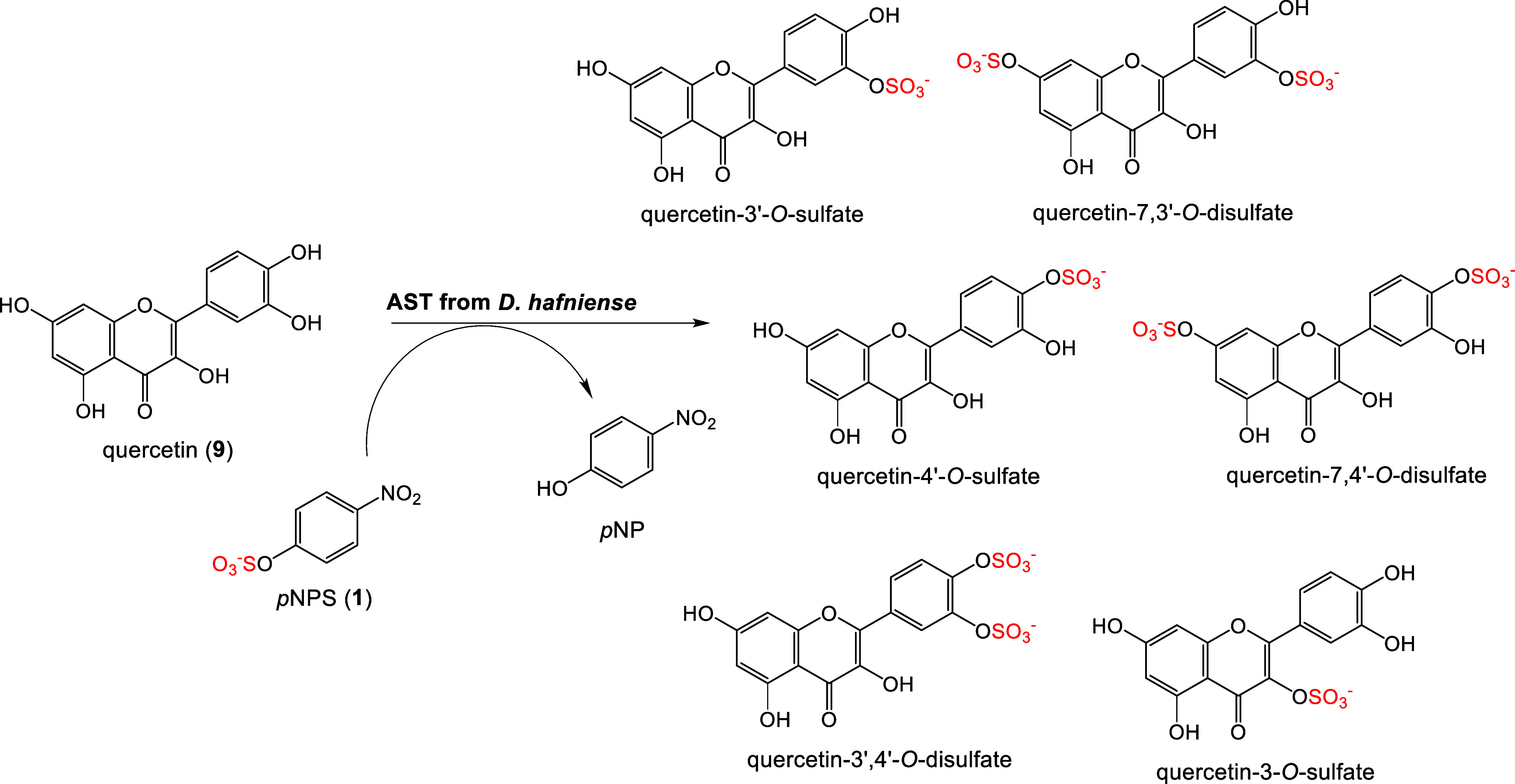
Sample sulfation of flavonoid quercetin (**9**) catalyzed by bacterial PAPS-independent aryl sulfotransferase from *Desulfitobacterium hafniense* affording identified sulfated products.^17^

Therefore, reliable synthetic pathways to sulfated metabolites of flavonoids are of great utility for food, pharmaceutical, and biomedical research. While conventional chemical sulfation methods are often complicated, require multiple process steps, and may yield unstable final products, enzymatic sulfation is a simpler alternative for this purpose.^12^ Recent studies have focused on 3′-phosphoadenosine 5′-phosphosulfate (PAPS)-independent bacterial aryl sulfotransferases (ASTs; EC 2.8.2.1). These enzymes enable the transfer of sulfate groups from phenolic donors to acceptors without PAPS mediation (Figure 1) and offer new perspectives for sustainable synthesis of sulfated bioactive compounds.^13−16^

The best-known sulfate donors for ASTs comprise *p-*nitrophenyl sulfate (*p*NPS, **1**) and 4-methylumbelliferyl sulfate (MUS),^18−20^ which are inexpensive, more readily available, and more stable than PAPS. While MUS is mainly used for kinetic determination and enzyme assays,^21,22^*p*NPS is suitable for the sulfation of polyphenolic compounds such as flavonoids in good yields.^23,24^ These differing applications are also related to the different catalytic efficiencies of these substrates.^16^

Typical representatives of flavonoids differ mainly in the number and positions of –OH residues on their scaffold, which determines their properties and function. The absorption of flavonoids into the human bloodstream occurs in the gut, where the intestinal microbiota metabolize some compounds mostly to phenolic acids.^25^ These acids also undergo phase II biotransformation and are putative substrates for ASTs; these enzymatic reactions are generally known to be regioselective and specific for a particular substrate. Their specificity and catalytic efficiency can change from one substrate to another.

Importantly, the efficiency of the transferase action of ASTs depends on both the donor and the acceptor. Reactions using the same sulfate donor **1** proceed differently with different acceptors. Therefore, finding the best match between the donor and acceptor is vital for the subsequent production of sulfated metabolites of flavonoids or other phenolic compounds.

**Figure 2:**
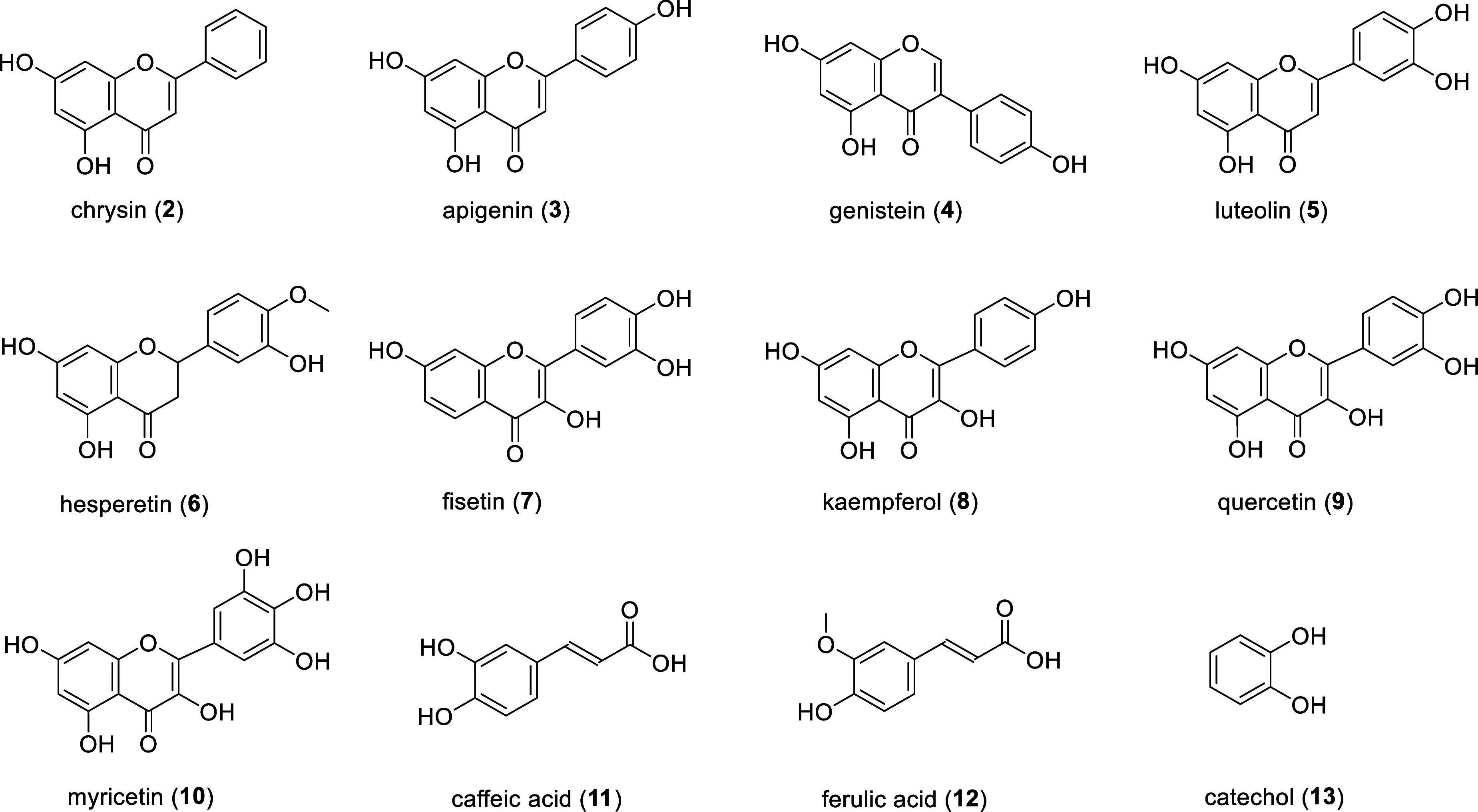
Selected sulfate acceptors for new recombinant ASTs. Chrysin (**2**), apigenin (**3**), genistein (**4**), luteolin (**5**), hesperetin (**6**), fisetin (**7**), kaempferol (**8**), quercetin (**9**), myricetin (**10**), caffeic acid (**11**), ferulic acid (**12**), and catechol (**13**).

This study presents a library of novel AST enzymes as sulfation tools and explores their potential in sulfating polyphenols, particularly flavonoids (Figure 2). The new ASTs showed a broader substrate specificity and greater efficiency than the previously published enzymes. Additionally, we demonstrated the synthetic utility of the novel library by synthesizing a previously unpublished series of kaempferol (**8**) sulfates.

## Materials and Methods

### Materials

*p*-Nitrophenyl sulfate (**1**), phenol, catechol (**13**), and genistein (**4**) were purchased from Sigma-Aldrich (Merck KGaA, Darmstadt, Germany). Chrysin (**2**) and fisetin (**7**) were purchased from Thermo Scientific Chemicals (Thermo Fisher Scientific, Waltham, Massachusetts, USA). Apigenin (**3**) and hesperetin (**6**) were provided by Biosynth Ltd. (Compton, Berkshire, UK). Luteolin (**5**), kaempferol (**8**), quercetin (**9**), and myricetin (**10**) were from abcr GmbH (Karlsruhe, Germany). Caffeic (**11**) and ferulic (**12**) acids were purchased from Acros Organics (Thermo Fisher Scientific, Waltham, Massachusetts, USA). Chemicals for growth media (tryptone and yeast extract) were supplied by Oxoid (Thermo Fisher Scientific, UK). Tris, glycine, and phenylmethylsulfonyl fluoride (PMSF) from VWR Chemicals. Acetone, ethyl acetate, ethanol, and methanol were purchased from VWR International (Avantor, Inc., Radnor, Pennsylvania, USA). If not specified otherwise, all other chemicals came from Lach-Ner (Neratovice, CZ). All chemicals and solvents used were of analytical grade. His Trap column was obtained from Cytiva (Chicago, Illinois, USA). Sephadex LH-20 gel was purchased from GE Healthcare Bio-Sciences (Uppsala, Sweden).

### High-Performance Liquid Chromatography Analysis

The screening of the product formation was performed on a Shimadzu Prominence LC analytical system consisting of a Shimadzu LC-20AB binary high-performance liquid chromatography (HPLC) pump, a Shimadzu CTO-20A column oven, a Shimadzu SIL-20A HT cooling autosampler, and a Shimadzu SPD-20MA diode array detector (all Shimadzu, Kyoto, Japan). The data were analyzed using the Shimadzu Lab Solution program (version 5.97 SP2). The formation of *p*NP was observed at 316 nm. The reaction mixtures were analyzed using a tempered (45 °C) HPLC column Kinetex 5 μm PFP (pentafluorophenyl), 150 × 4.6 mm (Phenomenex, USA), with a PFP guard column (4 × 3 mm; Phenomenex, USA) and a flow rate of 0.6 mL min^–1^ by gradient elution of mobile phases **A**: 10 mM ammonium acetate/0.1% HCOOH, and **B**: 100% methanol. Two different methods of analysis were used. The gradient elution method for flavonoids **2**–**6**, and **8**–**10** was as follows: 0 min 40% **B**, 0–20 min 40–72% **B**, 20–21 min 72–40% **B**, 21–25 min 40% **B** for column equilibration. The gradient elution method for fisetin (**7**), phenolic acids **11**–**12**, and catechol (**13**) was as follows: 0 min 20% **B**, 0–20 min 20–50% **B**, 20–21 min 50–20% **B**, 21–25 min 20% **B** for column equilibration.

### Liquid Chromatography-Mass Spectrometry Analysis

Liquid chromatography-mass spectrometry (LC-MS) analyses were performed using a Shimadzu Prominence LC analytical system comprising a Shimadzu CBM-20A system controller, a Shimadzu LC-20AD binary HPLC pump, a Shimadzu CTO-10AS column oven, a Shimadzu SIL-20ACHT cooling autosampler, and a Shimadzu SPD-20MA diode array detector (Shimadzu, JP). The samples were dissolved in water (30 μL) and centrifuged.

LC-MS analyses of sulfated compounds **2**–**12** were performed on a Kinetex PFP column (150 × 4.6 mm, 5 μm) preceded by security guard cartridge (4 × 3.0 mm, Phenomenex, USA) with gradient elution (**A**: 10 mM ammonium acetate/0.1% HCOOH, **B**: MeOH/0.1% HCOOH). For sulfated compounds **2**–**10** the gradient was as follows: 40% **B** for 0 min, 40–72% **B** for 0–20 min, 72–40% **B** for 20–21 min, and 40% **B** for 21–24 min for column equilibration; for the reactions with compounds **11**–**12** the gradient was as follows: 20% **B** for 0 min, 20–50% **B** for 0–20 min, 50–20% **B** for 20–21 min, and 20% **B** for 21–24 min for column equilibration; for compound **13** the gradient was: 20% **B** for 0 min, 20–35% **B** for 0–11 min, 35–20% **B** for 11–12 min, and 20% **B** for 12–15 min for column equilibration; flow rate was 0.6 mL min^–1^, 45 °C, injection volume 1 μL. The MS-ESI parameters were as follows: positive and negative mode; ESI interface voltage, 4.5 kV, −3.5 kV; detector voltage, 1.15 kV; nebulizing gas flow, 1.5 mL min^–1^; drying gas flow, 15 mL min^–1^; heat block temperature, 200 °C; the temperature of desolvation line pipe, 250 °C, interface temperature 350 °C, SCAN mode 80–500 *m*/*z*. The chromatograms were analyzed using the software LabSolutions ver. 5.75 SP2 (Shimadzu, Kyoto, Japan).

### Liquid Chromatography with High-Resolution Mass Spectrometry Analysis

Liquid chromatography with high-resolution mass spectrometry (LC-HRMS) with an electrospray ion source was used to analyze compounds **14**–**17**. The LC Agilent Infinity II system was used with a ZORBAX Eclipse Plus C18 column (1.8 μm, 3.0 × 50 mm) and a precolumn of the same type, both heated to 40 °C. Sample (2 μL) separation was performed in a binary gradient with 100% H_2_O (A) and 100% methanol (B) and the gradient method was set to a 0.4 mL min^–1^ flow rate. The method consisted of an isocratic phase (0–0.5 min, 5% B), followed by a gradient phase 5–100% B (0.5–5 min), and another isocratic phase with 100% B (5–13 min). Finally, the initial conditions (5% B) were restored over 3 min.

Before injecting the next sample, the column was equilibrated with the initial conditions for 5 min. The ion source was operated in negative ionization mode and the mass spectrometer (Agilent 6546, qTOF) had the following settings: drying gas temperature and flow at 250 °C and 8 L min^–1^, sheath gas temperature and flow at 400 °C and 12 L min^–1^, nebulizer pressure at 35 psi, capillary voltage at 3.5 kV, fragmentor at 140 V, skimmer at 65 V, Oct 1 RF Vpp at 750 V, mass range of 50–1700 *m*/*z*, and acquisition rate of 2 spectra/s. Data analysis was performed with Agilent MassHunter Qualitative Analysis 10.0 software.

### Nuclear Magnetic Resonance Analysis

Nuclear magnetic resonance (NMR) spectra of kaempferol **8** and its sulfates **14**–**17** were acquired on a Bruker Avance III 700 MHz spectrometer (Bruker BioSpin, Rheinstetten, Germany) in DMSO-*d*_6_ at 30 °C. ^1^H NMR, ^13^C NMR, HSQC, and HMBC experiments were performed using standard manufacturer’s software (TopSpin 3.5, Bruker BioSpin, Rheinstetten, Germany). ^1^H NMR and ^13^C NMR spectra were referenced using the solvent residual signals in DMSO-*d*_6_ (δ_H_ 2.499 ppm, δ_C_ 39.46 ppm). A two-parameter double-exponential Lorentz-Gauss function was applied for ^1^H to improve resolution. Line broadening (1 Hz) was used to get a better ^13^C signal-to-noise ratio. The assignment of proton spin systems was transferred to carbons by the HSQC experiment. The HMBC experiment provided the assignment of quaternary carbons and joined partial structures together. The sulfate position was determined using the upfield shifted signal of the substituted carbon and the downfield shifted signals of adjacent carbons compared to the signals of kaempferol.

### Design and Preparation of Plasmids

The genes encoding for putative ASTs were selected based on a previously published phylogenetic tree.^16^ The gene constructs encoding for putative ASTs, *e.g.*, *Dh*AST from *Desulfitobacterium hafniense, Ds*AST from *Desulfosporosinus* sp., *Dac*AST from *Desulfosporosinus acididurans, Dal*AST from *Desulfofalx alkaliphila*, *Nm*AST from *Niameybacter massiliensis*, *Hh*AST from *Hungatella hathewayi*, *Sb*AST from *Salmonella bongori*, *Sh*AST from *Shewanella oneidensis*, and *Cf*AST from *Campylobacter fetus* (GenBank accession nos: WP_015263010.1; WP_034601251; WP_047811757.1; WP_031517842; WP_053983873.1; WP_025529179.1; WP_038392742; WP_011073628.1 and WP_038452934, respectively) were designed in our laboratory and synthesized by Generay (China). Additionally, the gene construct of *Ec*AST from *Escherichia coli* (GenBank ID: AAN82229) was prepared for comparison. All gene constructs for the expression of the above ASTs with a hexahistidine tag at the C-terminus were inserted into the pET-26b(+) plasmid (*Nco*I/*Sac*I restriction sites) with kanamycin resistance as previously described for *Dh*AST (GenBank: ACL21750).^16^ Sequence analysis of the new plasmids (SEQme, Dobříš, Czech Republic) confirmed the correct subcloning.

### Expression and Purification of Putative ASTs

The obtained plasmids were used for the transformation of *E. coli* BL21(DE)pLysS cells, and protein expression was performed as previously described^16,23^ in 4 × 600 mL of LB medium with 0.1 mM kanamycin under the induction with isopropyl β-d-thiogalactopyranoside (IPTG). The next day after induction, the medium was centrifuged (4500 × g, 20 min) and the supernatant was discarded. The harvested biomass was then resuspended in 0.1 M Tris-glycine buffer pH 8.9 supplemented with 1% phenylmethylsulfonyl fluoride (PMSF) for protease inhibition and sonicated on ice at 80% amplitude (6 cycles × 2 min with 2 min pauses). Enzyme activity was measured, and the lysates exhibiting a detectable activity were processed as published previously:^16^ after dilution (1:5) with loading buffer (20 mM phosphate/500 mM NaCl/50 mM imidazole pH 8.5 for *Dh*AST, *Ds*AST, and *Dal*AST; 20 mM phosphate/500 mM NaCl/100 mM imidazole pH 8.5 for *Ec*AST, *Sb*AST, and *Cf*AST) and purified by ion metal affinity chromatography (IMAC) on a HisTrap column (5 mL, GE Healthcare, USA). Enzymes were eluted with a gradient (60 mL) of 0–100% elution buffer (20 mM phosphate/500 mM NaCl/100 mM imidazole pH 8.5 for *Dh*AST, *Ds*AST, and *Dal*AST; 20 mM phosphate/500 mM NaCl/500 mM imidazole pH 8.5 for *Ec*AST, *Sb*AST, and *Cf*AST) at a flow rate of 2 mL min^–1^. The eluted fractions were analyzed by SDS-PAGE (12% gel) and fractions containing an active purified enzyme were pooled together. Then, ASTs from Cluster I (*Ec*AST, *Sb*AST, and *Cf*AST) were dialyzed against 7 L of 0.1 M Tris-glycine buffer pH 8.9, filtered (0.22 μm), diluted (1:6) with 0.1 M Tris-glycine buffer pH 8.9, and gradually centrifuged (4000 × g, 15 min). ASTs from Cluster II (*Dh*AST, *Dal*AST, and *Ds*AST) were twice dialyzed against 7 L of 0.1 M Tris-glycine buffer pH 8.9, to remove remaining imidazole, filtered (0.22 μm), and then concentrated by centrifugation. The protein concentration was determined spectrophotometrically using the Bradford method (calibrated for IgG).^26^

### Enzyme Activity Assay

The AST activity was determined spectrophotometrically by measuring the release of *p*-nitrophenol (*p*NP; **1**) at 417 nm and 30 °C as published earlier.^16^ The reaction contained 5 mM *p*NPS, and 5 mM acceptor (phenol or catechol **13**) in 100 mM Tris-glycine buffer pH 8.9. The assay was initiated by adding 50 μL of a suitably diluted enzyme and the release of *p*NP was monitored continuously (Tecan Sunrise Absorbance Microplate Reader, Switzerland) for 5 min. One unit (U) of enzyme activity is defined as the amount of enzyme catalyzing the formation of 1 μmol of *p*NP per minute under the given conditions. The nonenzymatic release of *p*NP (negative control) was confirmed to be negligible.

### Biochemical Characterization of Aryl Sulfotransferases

As a part of the biochemical characterization of the newly produced enzymes, the thermostability and pH optimum were determined. The enzymatic activity of *Ds*AST was measured continuously at the respective temperature (15–80 °C) and the released *p*NP was detected at 417 nm. For the enzymes *Dal*AST, *Ec*AST, *Sb*AST, and *Cf*AST, which were more resistant to high temperatures and could show false positive results, the temperature profile was determined using the end point method, where an enzyme assay (250 μL) was performed at the respective temperature (15–85 °C) for 5 min in an Eppendorf microtube. Reactions were stopped by adding 20 μL (or 50 μL for *Cf*AST) of 1 M NaOH. Then, 50 μL of the stopped reaction mixture was added to 100 μL of 0.1 M Na_2_CO_3_ in a 96-well microtiter plate and the amount of the released *p*NP was calculated according to the respective calibration curves. When measuring pH optimum, the reactions were carried out in a discontinuous assay setup at the respective pH (5–13) at 30 °C and terminated by heating (99 °C for 5 min) with *Ds*AST. Reactions with *Ec*AST, *Sb*AST, and *Dal*AST were stopped by adding 20 μL (or 50 μL for *Cf*AST) of 1 M NaOH due to the high thermostability of the enzymes and measured as described above. The stability of the enzymes was tested in selected combinations of temperature (30–40 °C) and pH (7–9). The enzyme activity of the incubated samples was monitored for 48 h by assaying regular enzyme aliquots for their activity in the standard continuous assay. To test the stability of the enzymes in relevant organic solvents, the enzymes were incubated in 5% *v*/*v* DMSO, 10% *v*/*v* DMSO, or 20% *v*/*v* acetone. Enzyme activity was measured in the standard continuous assay and monitored for 48 h.

The kinetic parameters of the enzymes were also determined as a part of their biochemical characterization. The kinetic parameters were measured in 96-well microtiter plates at 30 °C with donor concentrations (*p*NPS, **1**) in a range of 0.02–50 mM and phenol or catechol (5–200 mM) as an acceptor. After adding the enzyme, the enzyme activity was measured continuously at 417 nm to detect the release of *p*NP. The kinetic parameters were calculated using nonlinear regression tools in ChemPad Prism v8 software (GraphPad Software, Boston, MA).

### General Procedure for Analytical Sulfation

Ten mg of substrate **2**–**9** (1 eq) were dissolved in DMSO (300 μL). Then, sulfate donor **1** (1.5 eq) and 50 mM Tris-glycine buffer pH 8.9 were added to a final reaction volume of 4 mL. The reactions were initiated by adding the purified enzyme (0.25 U mL^–1^ in the reaction mixture). The reactions ran for 48 h and were stopped by heating (99 °C for 5 min). Aliquots (100 μL) were taken during the reactions for TLC analysis (ethyl acetate/methanol = 9:1, *v*/*v*) at *t* = 0, 10, 30, 60 min, then at 2, 4, 24, and 48 h. Samples taken at 0, 2, 24, and 48 h of the reaction were analyzed by HPLC. The formation of *p*NP and sulfated products was confirmed at 48 h by LC-MS. The reaction conversion was determined as the formation of *p*-nitrophenol released from the *p*NPS donor, to directly monitor the enzyme function. We observed no noticeable degradation of flavonoid acceptor during the monitored time period. None of the detected products were found in the blank reactions.

**Table 1:** Kinetic Parameters of Recombinant ASTs with *p*NPS (**1**) as a Sulfate Donor and Phenol/Catechol as Acceptors

		phenol acceptor			catechol acceptor		
	enzyme	*K*_M_ [mM]	*k*_cat_ [s^–1^]	*k*_cat_/*K*_M_ [L mmol^–1^ s^–1^]	*K*_M_ [mM]	*k*_cat_ [s^–1^]	*k*_cat_/*K*_M_ [L mmol^–1^ s^–1^]
Cluster II	*Dh*AST^a^	0.34 ± 0.03	14.5 ± 0.7	42	2.8 ± 0.3	83 ± 6	30
	*Ds*AST	0.57 ± 0.02	32.5 ± 0.4	57	1.2 ± 0.1	3.3 ± 0.1	3.0
	*Dal*AST	0.034 ± 0.002	0.705 ± 0.007	21	0.36 ± 0.16	9.1 ± 0.1	25
Cluster I	*Ec*AST	0.71 ± 0.03	41.1 ± 0.5	57	0.54 ± 0.02	36.2 ± 0.4	67
	*Sb*AST	8.6 ± 0.4	37.9 ± 0.5	4.4	1.3 ± 0.9	30.3 ± 0.8	23
	*Cf*AST	0.189 ± 0.001	5.05 ± 0.07	26	1.14 ± 0.09	16.4 ± 0.4	14

a Adopted from our previous publication.^16^

**Table 2:** Sulfation of Flavonoids **2**–**10** and Phenolic Acids **11**, **12** by ASTs

	sulfation acceptors											
enzyme	number of regioisomers formed (monosulfates/disulfates)^a^ conversion of *p*NPS donor, releasing *p*NP [%]											
	2	3	4	5	6	7	8	9	10	11	12	13
*Dh*AST	1/0 2%	2/0 25%	1/0 39%	4/0 56%	2/0 20%	1/0 7%	1/0 46%	1/0 19%	1/0 24%	2/0 38%	1/0 44%	1/0 59%
*Ds*AST	1/0 1%	1/1 12%	1/0 10%	3/0 24%	2/0 5%	2/0 21%	1/0 28%	0/0^b^7%	0/0^b^3%	1/0 4%	0/0^b^7%	1/0 62%
*Dal*AST	2/0 9%	2/1 50%	1/1 53%	3/1 72%	2/1 53%	2/1 68%	2/1 61%	2/1 72%	0/0^b^55%	2/0 60%	1/0 60%	1/0 63%
*Ec*AST	0/0 1%	1/0 15%	1/0 3%	3/1 50%	2/0 13%	3/1 31%	2/1 55%	2/0 21%	1/0 11%	1/0 2%	0/0^b^11%	1/0 62%
*Sb*AST	1/0 1%	2/1 22%	1/0 15%	4/0 38%	2/0 9%	2/0 27%	1/0 41%	1/0 14%	1/0 4%	2/0 15%	0/0^b^8%	1/0 60%
*Cf*AST	1/0 8%	2/0 20%	1/0 25%	1/0 66%	2/0 33%	1/0 77%	2/1 48%	1/0 58%	2/0 36%	2/0 60%	1/0 53%	1/0 62%

a Reactions were performed in 50 mM Tris-glycine buffer pH 8.9 (or in 100 mM buffer for substrates **11**–**12**) containing 7% *v/v* DMSO, 1 eq of acceptor, and 1.2 eq of sulfate donor pNPS. All reactions were initiated by adding 1 U of AST. After 48 h the reactions were stopped and analyzed for sulfated products by LC-MS. Sulfation and reaction conversions were monitored by HPLC by detecting the individual sulfates and measuring the released *p*NP, respectively. The retention times of individual regioisomers are listed in the Supporting Information, Table S2.

b Product degradation.

Enzymatic sulfation of myricetin (**10**): The reactions containing substrate **10** (10 mg, 7.9 mM) were performed as described in the General procedure under argon atmosphere to prevent spontaneous oxidation of the substrate. The rate of *p*NP formation was monitored by TLC and HPLC at *t* = 0, 2 h, then at 24, and 48 h. HPLC-MS analysis was performed at 48 h.

Enzymatic sulfation of caffeic acid (**11**) and ferulic acid (**12**): The reactions with substrates **11** and **12** (6.3 mg, 8.8 mM) were performed as described in the general procedure but with 100 mM Tris-glycine buffer pH 8.9 for better maintenance of reaction pH. Reactions were performed under argon atmosphere to prevent spontaneous oxidation of the substrate. The rate of *p*NP formation was monitored by TLC and HPLC at *t* = 0, 2 h, then at 24, and 48 h. HPLC-MS analysis was performed at 48 h.

Enzymatic sulfation of catechol (**13**): The reactions with substrate **13** (3.8 mg, 8.8 mM) were performed as described in the general procedure with **1** as a sulfate donor. The rate of *p*NP formation was monitored by TLC and HPLC at *t* = 0, 2 h, then at 24, and 48 h. HPLC-MS analysis was performed at 48 h.

### Sulfation of Kaempferol (**8**) by *Dal*AST in the Presence of Acetone

Kaempferol (**8**, 3.5 mM) was dissolved in 0.5 mL of acetone. The reaction mixtures were then prepared by adding sulfate donor **1** (4.2 mM) and 50 mM Tris-glycine buffer (pH 8.9) to a total reaction volume of 10 mL. Reactions were initiated by adding purified enzyme *Dal*AST or crude *Dal*AST from cell lysate (both 0.2 U mL^–1^ of the reaction mixture) or cell lysate of untransformed *E. coli* cells (with no measurable AST activity). Aliquots were taken at 10, 20, and 30 min, and then at 1, 2, 4, 24, and 48 h. The samples were analyzed using HPLC to compare the profile and the amount of formed products in both reactions.

### Preparative Sulfation of Kaempferol (**8**)

Kaempferol (**8**, 100 mg, 0.35 mmol) was dissolved in acetone (5 mL). Sulfate donor **1** (0.42 mmol) was dissolved in 50 mM Tris-glycine buffer pH 8.9, and the acceptor was added to a total reaction volume of 100 mL. The reaction was initiated by adding *Dal*AST cell lysate (0.5 U mL^–1^ of the reaction mixture). The sulfation ran at 30 °C with shaking for 24 h. The reaction was stopped by heating (5 min, 99 °C). Subsequently, the reaction mixture was treated as in previous publications.^16,24^ Acetone was removed under vacuum and reaction pH was adjusted to 7.5–7.7 using formic acid. Then, the reaction mixture was extracted with ethyl acetate (6 × 50 mL) to remove the residual *p*NP. The sulfation products were separated on a Sephadex LH-20 gel column (methanol/water = 80:20, *v*/*v*). Fractions containing the same product were pooled, freeze-dried, and characterized by HPLC, LC-MS, and NMR.

## Results and Discussion

### Production and Purification of Putative ASTs

To date, only a few characterized PAPS-independent bacterial aryl sulfotransferases have been published, but they already proved their efficiency in the sulfation of natural compounds like luteolin, naringenin, ampelopsin and other flavonoids including flavonolignans.^16,23,24,27−29^ Other ASTs were also able to sulfate aliphatic alcohols, parabens, and steroids.^13,30−33^ This study aims to establish a larger library of ASTs for future use. A diverse set of ASTs, differing in catalytic properties, substrate affinities, and stability in various media (pH, cosolvents, temperature), will be valuable for optimizing sulfation procedures to produce standards of human metabolites. So far, there has been virtually no information on the catalytic differences between the two aryl sulfotransferase clusters, in part because each cluster was represented only by a single characterized enzyme (Cluster I—ATS from *E. colivs* Cluster II—AST from *D. hafniense*). Eleven genes encoding for putative ASTs were selected from different bacteria from a phylogenetic analysis tree.^16^ Upon analyzing Cluster II, using the reference sequence of AST from *D. hafniense* (*Dh*AST, ACL21750.1),^13^ we selected the sequences encoding for ASTs from *Desulfitobacterium dichloroeliminans* (*Dd*AST, WP_015263010.1, 88% identity to *Dh*AST), *Desulfosporosinus* sp. HMP52 (*Ds*AST, WP_034601251.1, 84% identity to *Dh*AST), *D. acididurans* (*Dac*AST, WP_047811757.1, 77% identity to *Dh*AST), *Desulfofalx alkaliphila* (*Dal*AST, WP_031517842.1, 61% identity to *Dh*AST), *Niameybacter massiliensis* (*Nm*AST, WP_053983873.1, 53% identity to *Dh*AST) and *Hungatella hathewayi* (*Hh*AST, WP_025529179.1, 50% identity to *Dh*AST). Upon analyzing Cluster I using the reference sequence of the AST from *E. coli* CFT073 (*Ec*AST, AAN82229.1),^22^ we selected the sequences encoding for ASTs from *S. bongori* (*Sb*AST, WP_038392742.1, 88% identity to *Ec*AST), *S. oneidensis* (*Sh*AST, WP_011073628.1, 60% identity to *Ec*AST), and *C. fetus* (*Cf*AST, WP_038452934.1, 47% identity to *Ec*AST). All enzymes were expressed in *E. coli* BL21(DE3)pLysS as C-terminal His-tagged constructs. Before purification, enzyme activity was measured in cell lysates with *p*NPS (**1**) as a sulfate donor and phenol or catechol (**13**) as an acceptor. Based on this pre-screening, the enzymes *Sh*AST, *Hh*AST, *Nm*AST, *Dac*AST, and *Dd*AST were excluded from subsequent purification and characterization due to their negligible activity in the cell lysate before purification (total isolated activity lower than 2 U), low biomass yields (lower than 2.5 g of biomass per 1 L of medium) or low specific activity (phenol or catechol, the higher of them) after purification (lower than 1 U mg^–1^). Enzymes *Dh*AST, *Ds*AST, *Dal*AST, *Ec*AST, *Sb*AST, and *Cf*AST were purified to homogeneity in a single step by nickel ion affinity chromatography (Supporting Information, Table S1). The purity of the enzymes was assessed by SDS-PAGE and their molecular mass (ca. 70 kDa) matched the theoretical molecular mass calculated from the protein sequence (Supporting Information, Figure S1).

AST from *E. coli* CFT073 was previously produced, characterized, and crystallized as a wild-type enzyme by Malojčić group^22^ and we use it here as a benchmark of Cluster I ASTs. The purification process included, as published, dialysis of the cell lysate, followed by anion exchange chromatography, chromatography on hydroxyapatite, and finally size exclusion chromatography. The overall yield of all purification steps was 2 mg of purified enzyme per 1 L of bacterial culture. In this work, we produced and characterized this AST as a His-tagged construct at the C-terminus (*Ec*AST), and we could purify the protein in a single step using IMAC, achieving a final yield of 1.5 mg of a pure protein per liter of cell culture medium. *Dh*AST, the benchmark enzyme of Cluster II, has been previously published by our group as a His-tagged construct,^16^ but not all its properties have been described so far. Here, we provide a detailed characterization of the present ASTs to give a detailed picture of their properties for the sake of comparison and easier applicability.

### Biochemical Characterization of Novel ASTs

Novel recombinant ASTs were biochemically characterized, and their kinetic parameters were determined. The temperature profile of the enzymes lay in a similar interval with the highest activity in a temperature range of 40–55 °C. ASTs are alkaline enzymes with pH optima ranging from 8 to 11 for *Dh*AST, *Ds*AST, and *Sb*AST. *Cf*AST had a narrow pH profile with an optimum of pH 12. In contrast, *Ec*AST and *Dal*AST had broader pH profiles: the pH optimum of *Ec*AST ranged from pH 6 to 10, while it ranged from pH 7 to 10.5 in *Dal*AST (Supporting Information, Figures S2, S3). These data are comparable to those published previously for *Ec*AST from *E. coli* CFT073 and *Dh*AST.^13,22^

The stability of enzymes is a decisive factor for their successful application in biocatalysis. In some cases, enzymes cannot remain active for more than a few minutes under certain “optimal” conditions,^34^ such as temperatures higher than 50 °C, which often show as temperature optimum in a standard activity assay taking only several minutes. Therefore, the stability of the produced ASTs was tested at relevant combinations of pH and temperature. All enzymes were found to be stable and active for more than 24 h at 30–40 °C and pH 9. These parameters were chosen for the subsequent sulfation screening (35 °C, pH 9). One potential limitation is that most (poly)phenols are not soluble in aqueous solutions, which makes the analysis of the reaction mixtures challenging and may complicate/slow down the biotransformation due to low enzyme saturation by the substrate. To solubilize the substrates and enable accurate analysis, organic cosolvents are often added to the reactions, but some enzymes may be sensitive to these solvents and lose activity. Therefore, the stability of the present enzymes was determined in 5% *v/v* and 10% *v/v* DMSO as used in the screening reactions. Most of the ASTs retained at least 50% of their activity in these concentrations of DMSO, except for *Dh*AST, which lost more than 70% of its activity under these conditions after 48 h. This indicates lower stability of the His-tagged *Dh*AST compared to wild-type purified *Dh*AST,^13^ which remains active in 10% and 20% *v*/*v* DMSO. The presence of DMSO even increased the activity of *Ec*AST, *Sb*AST, *Dal*AST, and *Ds*AST during the measured period. Another commonly used organic solvent for flavonoids is acetone.^35^ This solvent is easier to remove from the reaction mixture for product purification than DMSO and is better suited for preparative reactions. Most of the tested enzymes retained their activity after 5 h of incubation in 20% *v/v* acetone, However, the activity of *Sb*AST, *Ds*AST, and *Ec*AST, decreased drastically to less than 20% during the same period.

As a part of the biochemical characterization, the kinetic parameters of the purified ASTs were determined with **1** as a sulfate donor (Supporting Information, Figures S4, S5). Phenol and catechol were selected as sulfate acceptors as usual in enzyme activity assays.^13,24,36^ Based on the results obtained (Table 1), some trends in the catalytic efficiency (*k*_cat_/*K*_M_) of the enzymes between the clusters could be identified. ASTs from Cluster I generally showed higher catalytic efficiency with catechol, while ASTs from Cluster II were more effective with phenol as a sulfate acceptor. For *Sb*AST and *Ds*AST, the difference between the acceptors is almost 10-fold. Within each cluster, we can see that the new ASTs have a higher affinity to the acceptors than the known, previously published ASTs. In Cluster I, the new enzyme *Ds*AST showed a higher catalytic efficiency with phenol (57 L mmol^–1^ s^–1^) than the reference enzyme *Dh*AST (42 L mmol^–1^ s^–1^). *Dal*AST had a lower catalytic efficiency than *Dh*AST with both acceptors, but still exhibited the highest affinity to these substrates of the cluster (*K*_M, phenol_ = 0.034 ± 0.002 mM, and *K*_M, catechol_ = 0.36 ± 0.16 mM), indicating its broad specificity and strong binding to these substrates.

In Cluster II, the benchmark enzyme *Ec*AST showed the highest catalytic efficiency (57 L mmol^–1^ s^–1^ with phenol and 67 L mmol^–1^ s^–1^ with catechol). However, one of the new ASTs, *Cf*AST, exhibited a higher affinity to phenol, like *Dal*AST, indicating stronger substrate binding.

### Screening of Sulfation with Polyphenol Acceptors

Several flavonoids (**2**–**10**) and two phenolic acids (**11**–**12**, Figure 2) were selected as acceptors for the screening of the sulfation abilities of new recombinant ASTs. The acceptor selection was based on the structural differences between the substrates (mostly flavones), consisting mainly in different numbers and/or positions of –OH groups. Hesperetin (**6**) was proposed due to its methyl residue on its B-ring next to the –OH group (Figure 2), and ferulic acid was selected for the same reason. Some of the sulfate acceptors, like luteolin (**5**), quercetin (**9**), and myricetin (**10**), have been previously sulfated by wild-type AST from *D. hafniense*.^24,28,37^ Sulfated compounds were produced and characterized, serving as reference products for new ASTs. Previously, we tested several alternative sulfate donors, such as 4-methylumbelliferyl sulfate, *m*- and *o*-nitrophenyl sulfate, and phenyl sulfate, with *Dh*AST,^16^ but their relatively inefficient performance did not match their high price. Additionally, *p*NPS (**1**) is commonly used as a sulfate donor in the literature, so we chose this donor as well. The reactions with the new ASTs were carried out using **1** as a sulfate donor, at a constant temperature (35 °C) and pH (8.9). Each reaction contained 1 U of the respective enzyme as determined in the standard activity assay; the analytical sulfation of catechol (**13**) served as a reference. The reaction mixtures were analyzed by HPLC for the formation of *p*NP and sulfated products. Sulfated products were detected by LC-MS after 48 h of the reaction.

Based on the results (Table 2), we could assess the ability of individual enzymes to sulfate phenolic compounds and produce a range of sulfated products. Most of the screened ASTs exhibited the highest conversion rates with the positive control acceptor catechol, reaching 60%, while the conversion rates with other acceptor substrates reached barely more than 30% after 48 h. In most reactions, the reaction stopped after 24 h, so no new *p*NP was detected at *t* = 48 h (Supporting Information, Figure S6). The lowest conversions were observed mainly with chrysin (**2**), probably due to the absence of hydroxyl groups (OH) on the B-ring, where sulfation primarily occurs. The sulfation on the A-ring is rare and usually happens as a secondary reaction during disulfate formation.^17,24^ Luteolin (**5**) and kaempferol (**8**) emerged as the most favorable substrates, as they consistently showed the highest conversion rates for all enzymes tested. Products were also detected in the reactions with phenolic acids **11** and **12**. In certain reactions, especially with ferulic acid (**12**), *p*NP was detected after 48 h without any product being detected by the LC-MS analysis. The negative control assay of *p*NPS (**1**) decomposition revealed no evidence of spontaneous *p*NP release or substrate hydrolysis. We hypothesize that this phenomenon, reminiscent of previous observations with monohydroxy phenolic acids, such as monohydroxyphenyl acetic acid,^38^ may suggest that while AST can cleave the sulfate group from the donor, it may not effectively attach it to the acceptor. Another possible explanation may be the degradation of the formed (unstable) sulfate product as, we suggest, may have happened in the reaction with *Dal*AST and **10**, where we observed a high reaction conversion (55%) with no detectable product after 48 h. To verify this hypothesis, LC-MS analysis of the reaction aliquot after 1 h (Supporting Information, Figures S7, S8) was performed and, indeed, the presence of the sulfated products was shown.

Among the ASTs tested, *Ds*AST exhibited the lowest efficiency in flavonoid sulfation with *p*NPS donor, closely followed by *Sb*AST, which achieved a maximum conversion of 40% with **8** as the best result. However, from the broader viewpoint of utility as a catalytic tool, the screening identified potentially very efficient AST candidates, with *Cf*AST and *Dal*AST exhibiting a broad substrate specificity compared with other ASTs. These ASTs produced more regioisomers and disulfates (seven disulfates detected with *Dal*AST), than previously known ASTs from *E. coli* (*Ec*AST) and *D. hafniense* (*Dh*AST). This points to the high sulfation efficiency of *Dal*AST with most substrates, since disulfates are secondary products formed primarily after the main reaction, monosulfation, reaches its limit. Hence, *Cf*AST and *Dal*AST exhibited a more pronounced regioselectivity and, in addition, the sulfation reactions had higher conversions. Based on these results, *Dal*AST was selected for the preparative synthesis of kaempferol sulfate due to its superior performance.

Previous studies have shown that *Dh*AST remains active and selective even in the crude form and effectively sulfates substrates using just cell lysate.^17,24,28^ To investigate this phenomenon with *Dal*AST, we compared the sulfation of kaempferol with both purified *Dal*AST and the crude enzyme in the cell lysate. The reactions were carried out under identical conditions (5% *v*/*v* acetone) and analyzed by HPLC to monitor product formation. The analysis showed that both reactions produced the same array of products. The same results were obtained after analyzing the screening reaction with purified *Dal*AST in DMSO after 24 h (Supporting Information, Figures S9–S11). Importantly, an analogous reaction performed with a lysate of untransformed *E. coli* cells cultivated under the same conditions yielded no products, which further confirms that even when using cell lysate, the observed products originated exclusively by the action of AST enzyme.

### Preparative Sulfation of Kaempferol (**8**)

**Figure 3:**
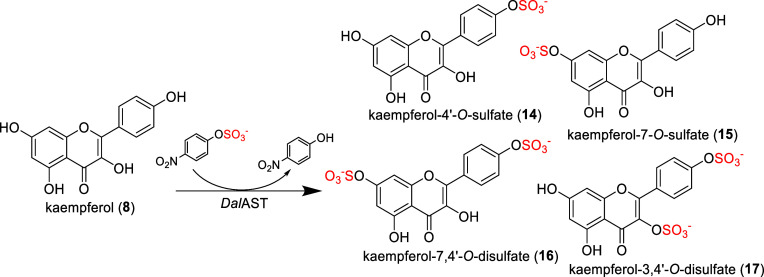
Sulfation of kaempferol (**8**) by *Dal*AST. Sulfated products kaempferol-4′-*O*-sulfate (**14**, 26 mg, 21% yield) and kaempferol-7,4′-*O*-disulfate (**16**, 20 mg, 13% yield) were isolated and structurally characterized. Sulfated products kaempferol-7-*O*-sulfate (**15**, isolated in a mixture with **14** in a ratio of 3/7), and kaempferol-3,4′-*O*-disulfate (**17**) were identified and their structure was assessed.

After sulfation screening, substrates **5** (luteolin) and **8** (kaempferol) were found to be the most suitable sulfate acceptors. Though luteolin sulfates were already described as products of *Dh*AST,^24,28^ there is no published report on an *in vitro* synthesis of kaempferol sulfate(s). Based on the results obtained, the preparative sulfation of **8** (kaempferol) was carried out with *Dal*AST using *p*NPS (**1**) as a donor. All the products were prepared in one reaction from 100 mg of kaempferol (**8**). After reaction completion, the reaction mixture (pH corrected to 7.5) was extracted with ethyl acetate to dispose of *p*NP, and then loaded on a Sephadex LH-20 column in 4/1, *v*/*v*, methanol/water mobile phase for product isolation. The sulfation of kaempferol (for the NMR data of **8** see Supporting Information, Tables S3, S8, and Figures S12, S13) yielded a battery of monosulfate regioisomers and disulfates (Figure 3), that we were able to structurally characterize: kaempferol-4′-*O*-sulfate (**14**), kaempferol-7-*O*-sulfate (**15**), kaempferol-7,4′-*O*-disulfate (**16**) and kaempferol-3,4′-*O*-disulfate (**17**). The major product of the reaction was kaempferol-4′-*O*-sulfate (**14**; 26 mg), isolated in a 20% yield and structurally characterized (Supporting Information, Table S4, Figures S14, S18). The second product, kaempferol-7,4′-*O*-disulfate (**16**), was isolated in the amount of 20 mg, corresponding to a 13% isolated yield (Supporting Information, Table S6, Figures S24–S27). The third product, kaempferol-7-*O*-sulfate (**15**), was detected in a mixture with kaempferol-4′-*O*-sulfate (**14**) in a molar ratio of 3/7 to **14**, corresponding to a *ca* 2% yield (Supporting Information, Table S5, Figures S19–S23). The fourth product, kaempferol-3,4′-*O*-disulfate (**17**), had a low purity after isolation but could still characterized by MS and NMR (Supporting Information, Table S7, Figures S29–S33).

The search for new ASTs revealed putative game changers in the field of flavonoid sulfation for possible applications in synthetic reactions. In this study, we have presented four new active ASTs with different sulfation abilities for selected flavonoids and phenolic acids. Two of the enzymes presented, *Dal*AST and *Cf*AST, were found to be more efficient and functional than the previously published enzymes in the regioselective sulfation of flavonoids, making them promising and effective tools for a straightforward production of analytical standards of sulfated metabolites. This is the first report on the isolated, and structurally characterized sulfated derivatives of kaempferol.

## Supporting Information Available

Supporting Information contains additional data, Tables, and Figures on the production and biochemical characterization of recombinant ASTs, on the screening of their sulfation abilities, and structural characterization of isolated sulfate products (PDF)

## Abbreviations

AST: aryl sulfotransferase
*Cf*AST: aryl sulfotransferase from *Campylobacter fetus*
*Dac*AST: aryl sulfotransferase from *Desulfosporosinus acididurans*
*Dal*AST: aryl sulfotransferase from *Desulfofalx alkaliphile*
*Dh*AST: aryl sulfotransferase from *Desulfitobacterium hafniense*
DMSO: dimethyl sulfoxide
*Ds*AST: aryl sulfotransferase from *Desulfosporosinus* sp.
*Ec*AST: aryl sulfotransferase from *E. coli*
*Hh*AST: aryl sulfotransferase from *Hungatella hathewayi*
HPLC: high-performance liquid chromatography
IMAC: immobilized metal ion affinity chromatography
LC-MS: liquid chromatography-mass spectrometry
MUS: 4-methylumbelliferyl sulfate
*Nm*AST: aryl sulfotransferase from *Niameybacter massiliensis*
PAPS: 3′-phosphoadenosine 5′-phosphosulfate
PFP: pentafluorophenyl
PMSF: phenylmethylsulfonyl fluoride
*p*NP: *p*-nitrophenol
*p*NPS: *p*-nitrophenyl sulfate
*Sb*AST: aryl sulfotransferase from *Salmonella bongori*
SDS-PAGE: sodium dodecyl sulfate-polyacrylamide gel electrophoresis
*Sh*AST: aryl sulfotransferase from *Shewanella oneidensis*
TLC: thin-layer chromatography
