# Supplementary material for: New Bacterial Aryl Sulfotransferases: Effective Tools for Sulfation of Polyphenols
Source: J Agric Food Chem. 2024 Oct 1;72(40):22208–16. doi: 10.1021/acs.jafc.4c06771 (PMC11468790; doi:10.1021/acs.jafc.4c06771)
Supplement: Supplementary file 1 — jf4c06771_si_001.pdf [file jf4c06771_si_001.pdf]

Supporting Information

New Bacterial Aryl Sulfotransferases: Effective Tools for Sulfation of Polyphenols

Katerina Brodsky[1,2], Barbora Petránková[1,3], Lucie Petrásková[1], Helena Pelantová[1], Vladimír Křen[1], Kateřina Valentová[1] and Pavla Bojarová[1,*]

[1] *Institute of Microbiology of the Czech Academy of Sciences, Vídeňská 1083, CZ-142 00 Prague, Czech Republic.*

[2] *Department of Biochemistry and Microbiology, University of Chemistry and Technology Prague, Technická 3, CZ-166 28 Prague, Czech Republic.*

[3] *Department of Genetics and Microbiology, Faculty of Science, Charles University, Albertov 6, CZ-128 43 Prague, Czech Republic*

** Corresponding author. bojarova@biomed.cas.cz; (+420) 296 442 360*

Contents

1. Production of recombinant ASTs
2. Biochemical characterization of recombinant ASTs
   2.1. pH Profiles of recombinant ATSs
   2.2. Temperature profiles of recombinant ASTs
   2.3. Kinetic parameters of recombinant ASTs
3. Screening of recombinant ASTs for the sulfation of flavonoid and phenolic acid acceptors
4. Structural characterization of isolated kaempferol sulfate products

1. Production of recombinant ASTs

Table S1. Production yields of recombinant ASTs

Enzyme	Biomass [g/L medium]	Total activity before purification [U]	Total Isolated protein [mg][a]	Specific activity [U/mg][b]	Yield [%]
*Dh*AST[c]	4.7	408	17.9	3.0	13
DdAST	4.2	18	70.6[c]	0.08	ND[e]
DsAST	4.9	1640	30	5.8	11
DacAST	4.8	63	9.8	0.9	15
DalAST	4.9	229	32.3	3.5	40
NmAST	2.1	0.35	ND	ND	ND
HhAST	3.4	1.2	ND	ND	ND
EcAST	3.6	259	6.5	29.4	73
SbAST	5.3	60	2.5	17.5	74
ShAST	1.8	0.26	ND	ND	ND
CfAST	4.2	135	19.7	5.7	83

[a]1.2 L of medium, [b] Phenol or catechol as acceptors – the higher activity; [c] previously published data doi: 10.1002/cssc.202201253 ; [d] could not be purified to homogeneity, contained other proteins; [e] ND - not detected

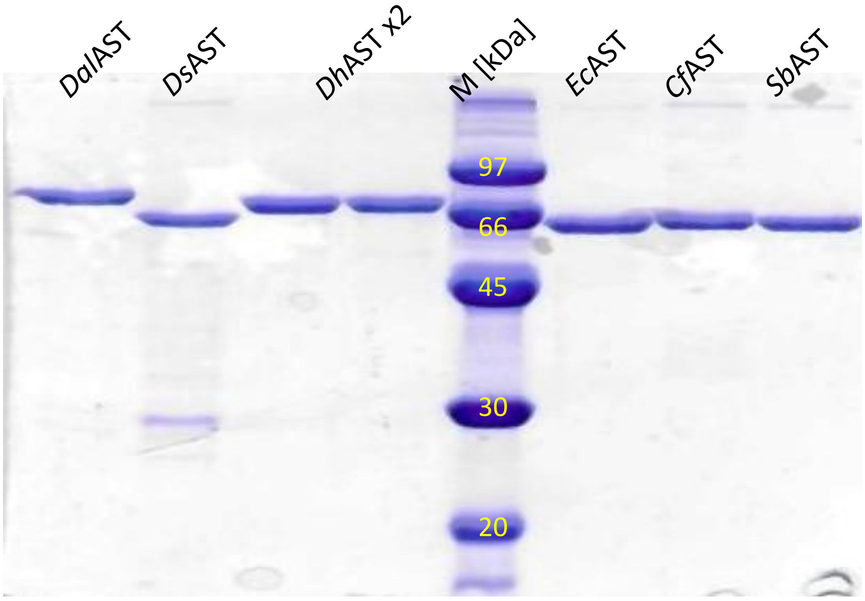

Figure S1. SDS-PAGE analysis of recombinant ASTs. Each lane shows the purity of the ASTs after IMAC purification. The enzymes have similar molecular weights (theoretical M_w calculated for the His-tagged constructs): *Dal*AST 73 kDa, *Ds*AST 73.3 kDa, *Dh*AST 73.5 kDa, *Ec*AST 68.5 kDa, *Cf*AST 68.8 kDa and *Sb*AST 68.7 kDa. M represents Low Molecular Weight Protein Marker (GE Healthcare, Chalfont St Giles, UK).

2. Biochemical characterization of recombinant ASTs

2.1. pH Profiles of recombinant ASTs

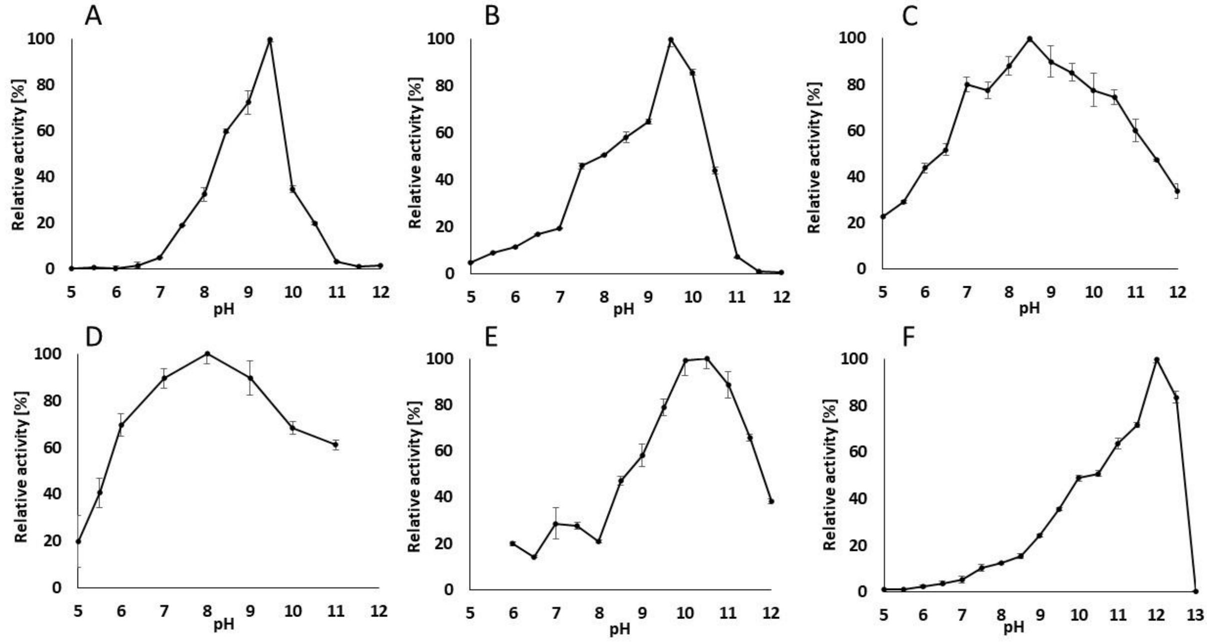

Figure S2. pH Profiles of recombinant ASTs. A – *Dh*AST[1], B – *Ds*AST, C – *Dal*AST, D – *Ec*AST, E – *Sb*AST, F – *Cf*AST.

2.2. Temperature profiles of recombinant ASTs

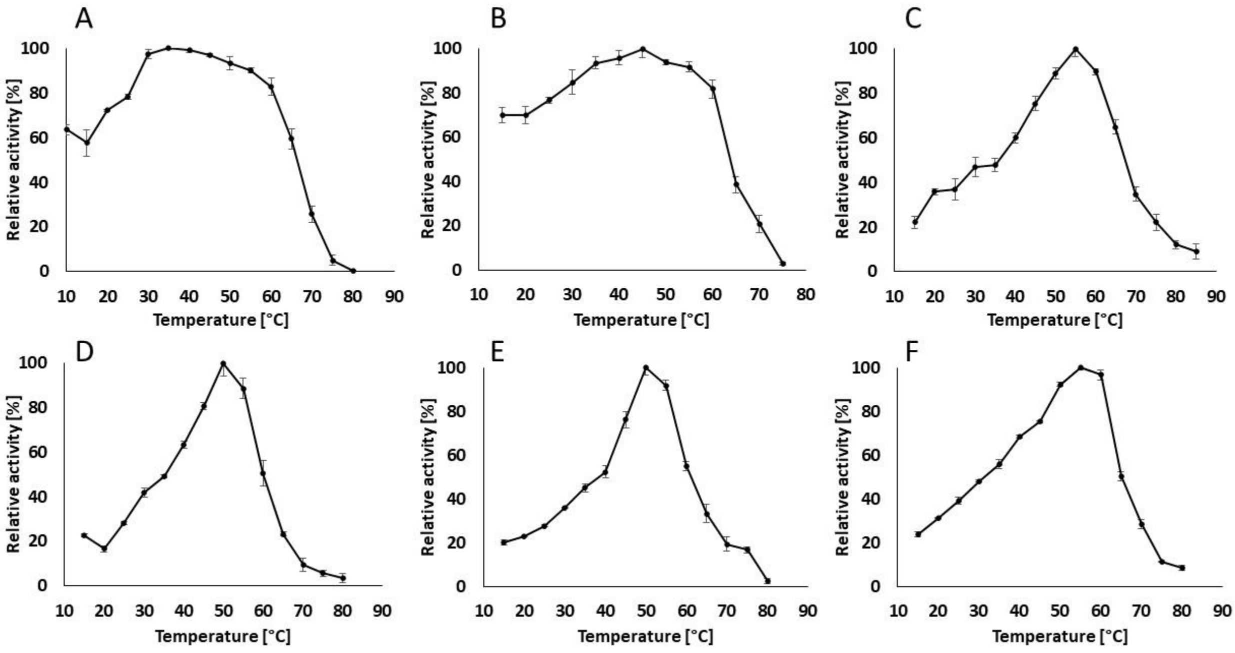

Figure S3. Temperature profiles of recombinant ASTs. A – *Dh*AST (published previously doi:10.1002/cssc.202201253), B – *Ds*AST, C – *Dal*AST, D – *Ec*AST, E – *Sb*AST, F – *Cf*AST.

2.3. Kinetic parameters of the recombinant ASTs

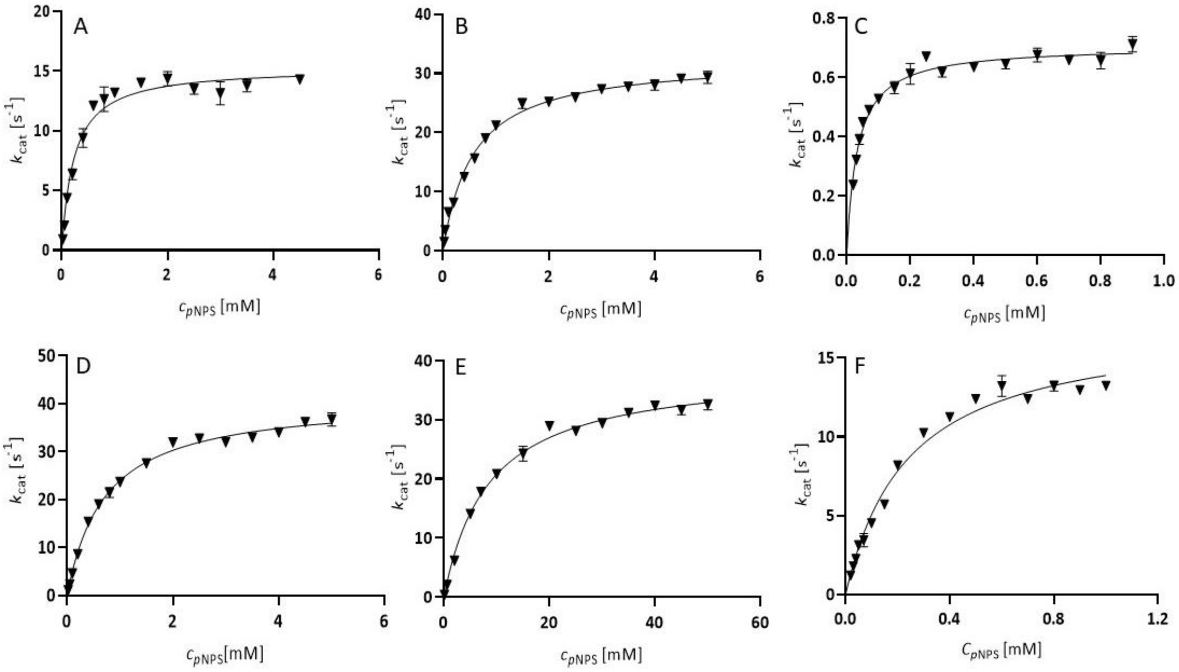

Figure S4. Kinetic parameters of recombinant ASTs for *p*NPS (**1**) as a sulfate donor with phenol as an acceptor in 100 mM Tris-glycine buffer. A – *Dh*AST (published previously 10.1002/cssc.202201253), B – *Ds*AST, C – *Dal*AST, D – *Ec*AST, E – *Sb*AST, F – *Cf*AST. Reactions were performed at 30 °C and pH optimum of the enzyme (*Dh*AST pH 9.5; *Ds*AST pH 9.5; *Dal*AST pH 8.5; *Ec*AST pH 8.0, *Sb*AST pH 10, *Cf*AST pH 11). The graphs were generated by the software Prism 8 (GraphPad, CA, USA).

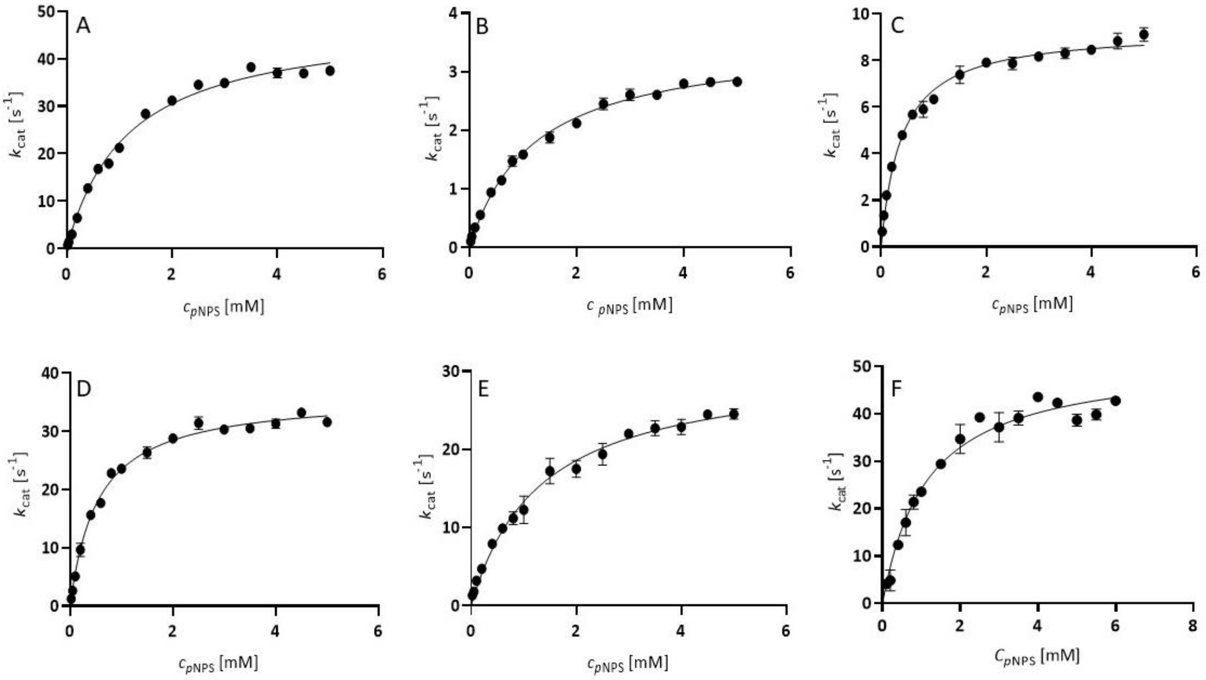

Figure S5. Kinetic parameters of recombinant ASTs for *p*NPS (**1**) as a sulfate donor with catechol as an acceptor in 100 mM Tris-glycine buffer. A – *Dh*AST (10.1002/cssc.202201253), B – *Ds*AST, C – *Dal*AST, D – *Ec*AST, E – *Sb*AST, F – *Cf*AST. Reactions were performed at 30 °C and pH optimum of the enzyme (*Dh*AST pH 9.5; *Ds*AST pH 9.5; *Dal*AST pH 8.5; *Ec*AST pH 8.0, *Sb*AST pH 10, *Cf*AST pH 11). The graphs were generated by the software Prism 8 (GraphPad, CA, USA).

3. Screening of recombinant ASTs for the sulfation of flavonoid and phenolic acid acceptors

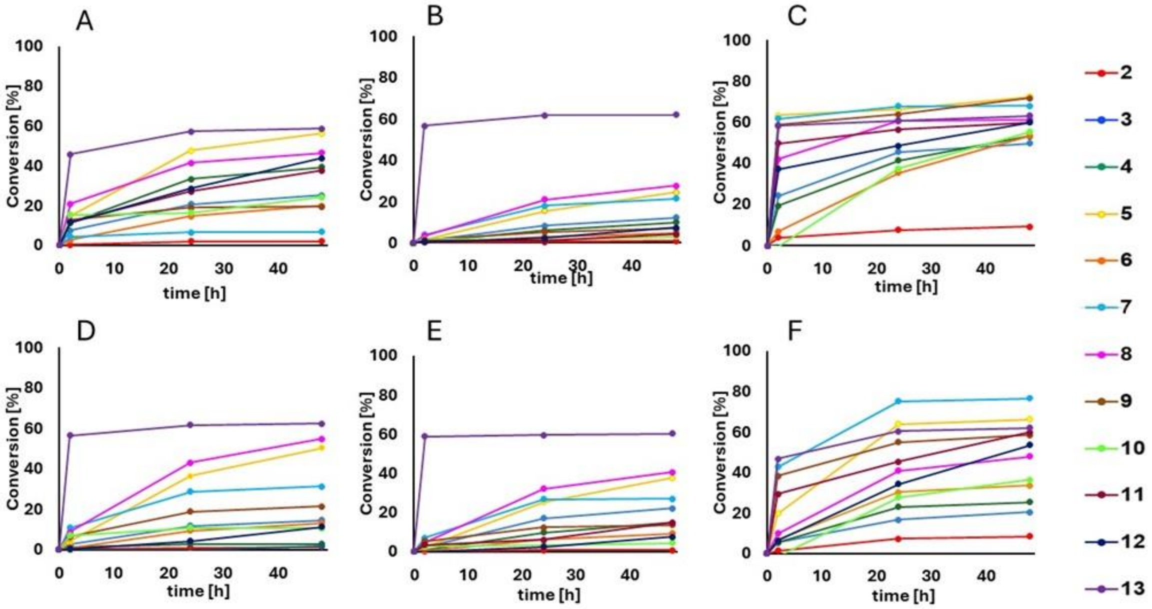

Figure S6. Conversion of *p*NPS (**1**) in sulfation screening reactions with substrates chrysin (**2**), apigenin (**3**), genistein (**4**), luteolin (**5**), hesperetin (**6**), fisetin (**7**), kaempferol (**8**), quercetin (**9**), myricetin (**10**), caffeic acid (**11**), ferulic acid (**12**) and catechol (**13**). A - *Dh*AST, B – *Ds*AST, C – *Dal*AST, D – *Ec*AST, E – *Sb*AST, F – *Cf*AST.

Table S2. Calculated and measured *m/z* and retention times of polyphenol sulfated products detected by LC-MS.

Substrate	Sulfated product[a]	Calculated *m/z*	Measured *m/z*	Retention times [min]
Chrysin (**2**)	mono-**S**	333.3	333	8.538, 15.865
Apigenin (**3**)	mono-**S**	349.3	349	12.403, 12.923
	di-**SS**	429.3	429	5.499, 6.271
Genistein (**4**)	mono-**S**	349.3	349	9.936
	di-**SS**	429.3	429	4.550
Luteolin (**5**)	mono-**S**	365.3	365	11.378, 12.195, 12.673, 13.207[b]
	di-**SS**	444.3	445	4.985
Hesperetin (**6**)	mono-**S**	381.3	381	8.120, 8.994[a]
	di-**SS**	461.4	461	3.749
Fisetin (**7**)	mono-**S**	365.3	365	6.275, 7.570, 8.201[b]
	di-**SS**	445.3	445	3.461
Kaempferol (**8**)	mono-**S**	365.3	365	10.424, 12.130[b]
	di-**SS**	445	445	4.822
Quercetin (**9**)	mono-**S**	381.3	381	9.666, 11.236, 12.250[b]
	di-**SS**	461.3	461	4.655
Myricetin (**10**)	mono-**S**	397.3	397	9.891, 11.435[b]
Caffeic acid (**11**)	mono-**S**	259.2	259	8.054, 8.417[b]
Ferulic acid (**12**)	mono-**S**	273.2	273	9.461
Catechol (**13**)	mono-**S**	189.3	189	4.337

Reaction conversions are indicated in Table 2 in the main text.

[a] "mono-S" indicates a monosulfate; "di-SS" indicates a disulfate; [b] regioisomers

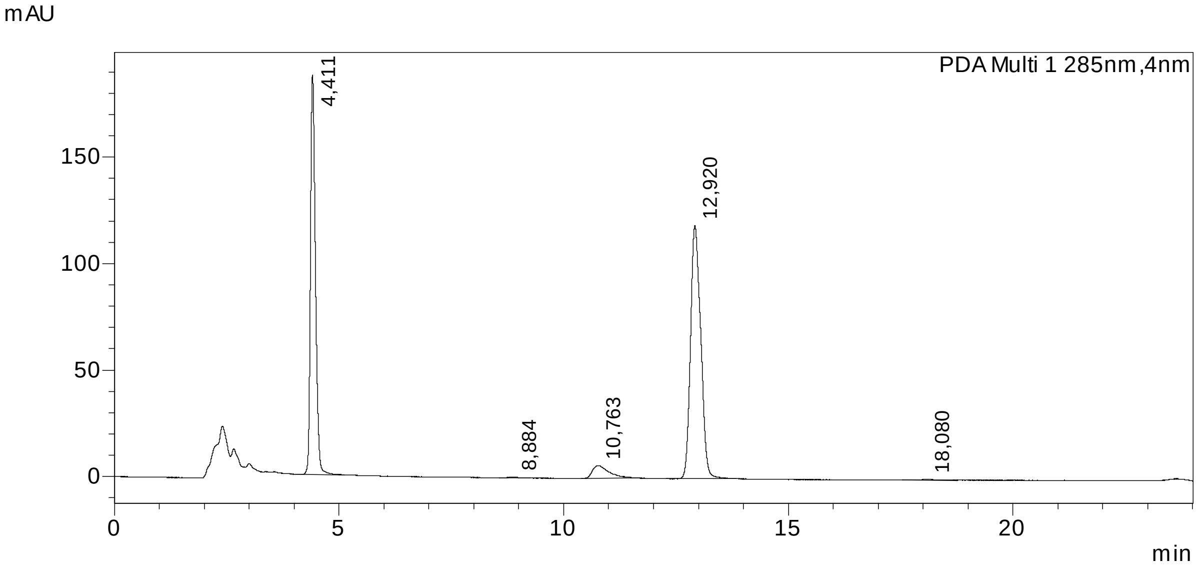

Figure S7. HPLC chromatogram of myricetin (**10**) sulfation catalyzed by *Dal*AST after 1 h of reaction. Retention times: myricetin (**10**) 18.08 min, released *p*NP 12.92 min, sulfation product myricetin sulfate 10.73 min, *p*NPS (**1**) 4.41 min.

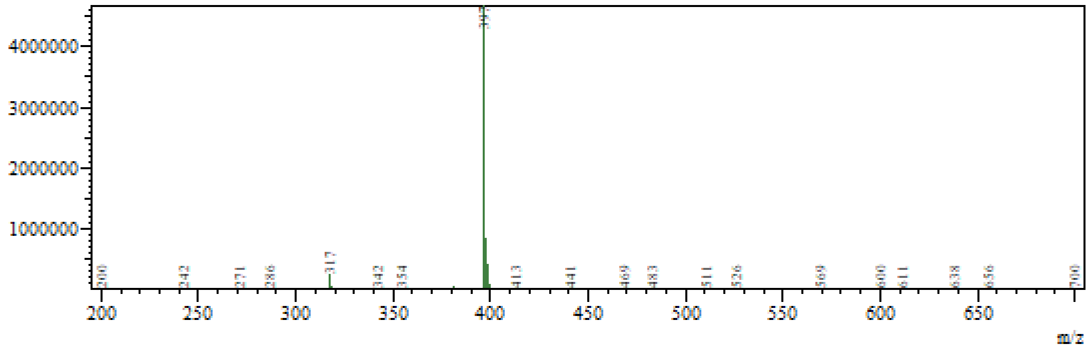

Figure S8. ESI-MS Spectrum of the product myricetin sulfate at 10.73 min in the negative mode: calculated *m*/*z* for [M-H]$^{-}$- 397.29, found 397.

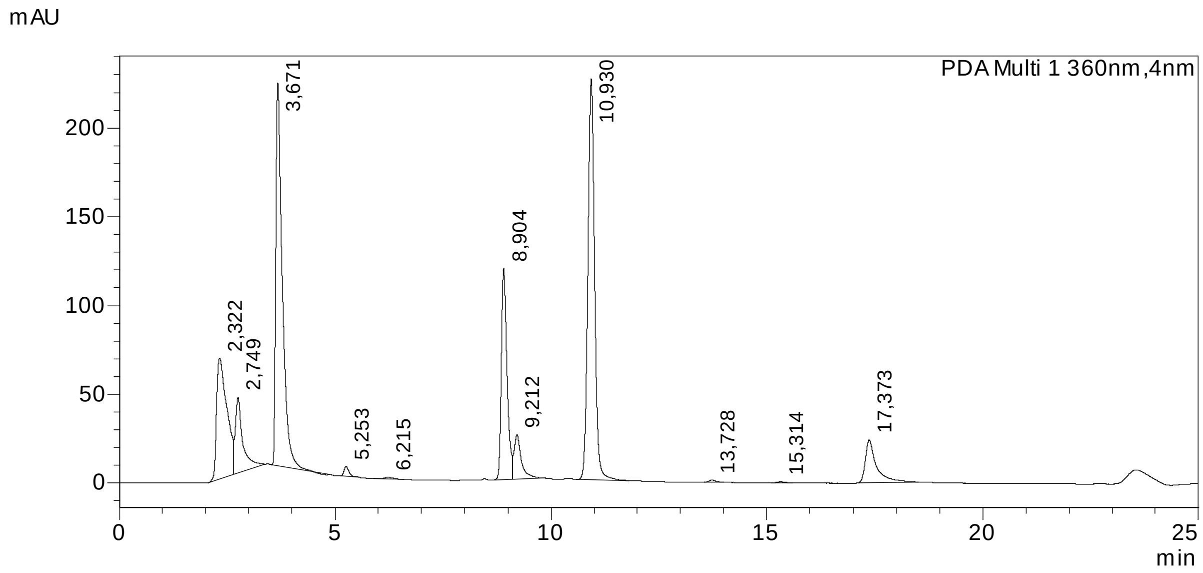

Figure S9. HPLC chromatogram of kaempferol (**8**) sulfation catalyzed by purified *Dal*AST after 24 h. The reaction mixture contained 7.5% *v*/*v* DMSO. Disulfate kaempferol isomers were detected at *t* = 2.3 and 2.7. Monosulfate kaempferol isomers were detected at *t* = 8.9 and 9.2 min. Residual *p*NPS was detected at *t* = 3.7 min, *p*NP at *t* = 10.9 min, and residual kaempferol at *t* = 17.4 min.

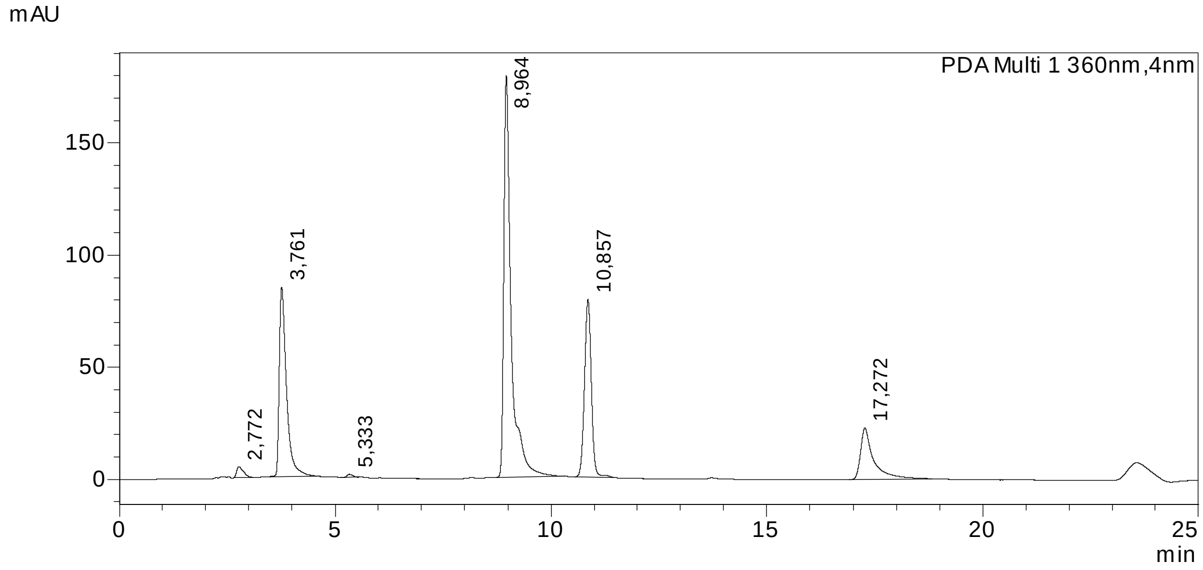

Figure S10. HPLC chromatogram of kaempferol (**8**) sulfation catalyzed by purified *Dal*AST after 24 h. The reaction mixture contained 5% *v*/*v* acetone. Disulfate kaempferol isomers were detected at *t* = 2.7. Monosulfate kaempferol was detected at *t* = 8.9 min. Residual *p*NPS was detected at *t* = 3.7 min, *p*NP *t* = 10.9 min, and residual kaempferol at *t* = 17.3 min.

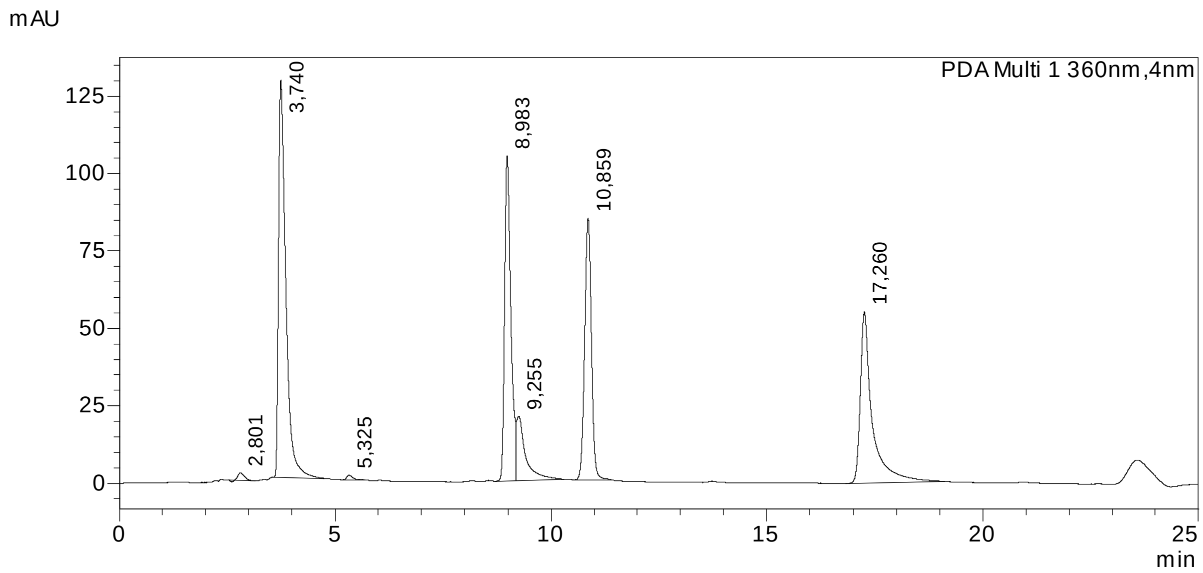

Figure S11. HPLC chromatogram of kaempferol (**8**) sulfation catalyzed by cell lysate containing *Dal*AST after 24 h. The reaction mixture contained 5% *v*/*v* acetone. Disulfate kaempferol isomers were detected at *t* = 2.8 min. Monosulfate kaempferol isomers were detected at *t* = 8.9 and 9.2 min, *p*NPS at *t* = 3.7 min, released *p*NP was detected at *t* = 10.9 min, and residual kaempferol at *t* = 17.3 min.

4. Structural characterization of isolated kaempferol sulfate products

Table S3. ^{1}H and ^{13}C NMR data for the starting compound kaempferol (**8**); 700.13 MHz for ^{1}H, 176.05 MHz for ^{13}C, DMSO-d_6, 30 °C.

Atom	δ_C	m.	δ_H	n_H	m.	*J* [Hz]
**2**	146.74	s	-	0		
**3**	135.56	s	-	0		
**4**	175.82	s	-	0		
**5**	160.63	s	-	0		
**6**	98.11	d	6.188	1	d	2.1
**7**	163.81	s	-	0		
**8**	93.38	d	6.433	1	d	2.1
**9**	156.10	s	-	0		
**10**	102.96	s	-	0		
ipso-	121.59	s	-	0		
ortho-	129.41[x]	d	8.039	2	m	Σ*J* = 9.0
meta-	115.35[x]	d	6.924	2	m	Σ*J* = 9.0
para-	159.10	s	-	0		
**5-OH**	-	-	12.468	1	s	
**3-OH**	-	-	9.347[a]	1	br s	
**7-OH**	-	-	10.072[a]	1	br s	
***para*-OH**	-	-	10.745[a]	1	br s	

[x] … 2C; [a] … might be interchanged

Structure of kaempferol with the numbering used for NMR characterization:

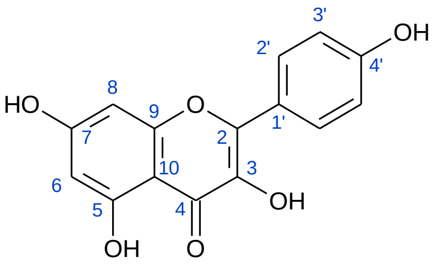

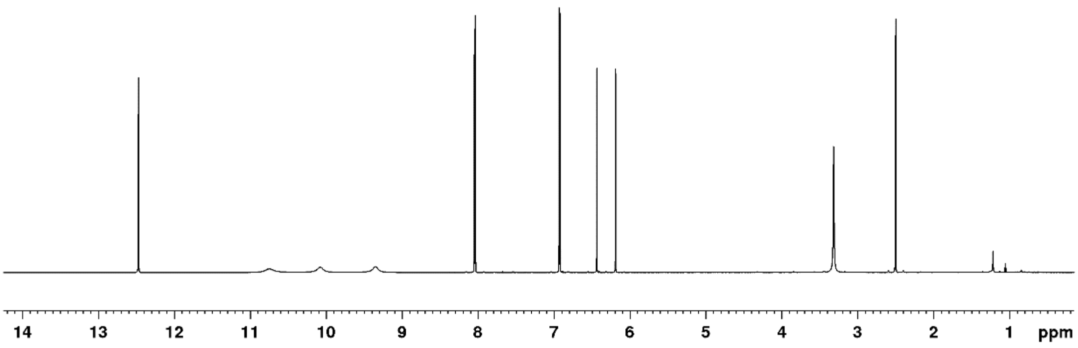

Figure S12. ^{1}H NMR Spectrum of compound **8** (700.13 MHz for ^{1}H, DMSO-d_6).

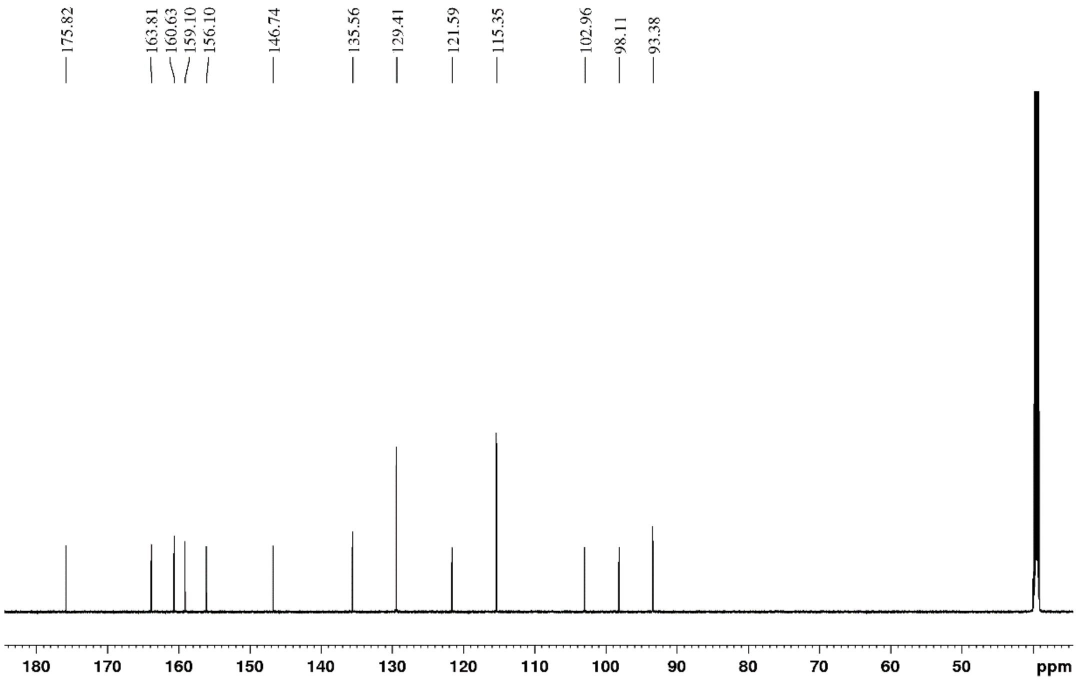

Figure S13. ^{13}C NMR Spectrum of compound **8** (176.05 MHz for ^{13}C, DMSO-d_6).

Table S4. ^{1}H and ^{13}C NMR data for the compound kaempferol-4'-*O*-sulfate (**14**); 700.13 MHz for ^{1}H, 176.05 MHz for ^{13}C, DMSO-d_6, 30 °C.

**Atom**	δ_C	**m.**	δ_H	n_H	m.	***J* [Hz]**
**2**	145.70	s	-	0		
**3**	136.31	s	-	0		
**4**	175.85	s	-	0		
**5**	160.60	s	-	0		
**6**	98.50	d	6.149	1	dd	2.0, 0.7
**7**	165.22	s	-	0		
**8**	93.61	d	6.401	1	m	
**9**	156.33	s	-	0		
**10**	102.60	s	-	0		
**1'**	125.36	s	-	0		
**2'**	128.38^x	d	8.081	2	m	Σ*J* = 9.0
**3'**	119.73^x	d	7.327	2	m	Σ*J* = 9.0
**4'**	154.82	s	-	0		

^{x} … 2C

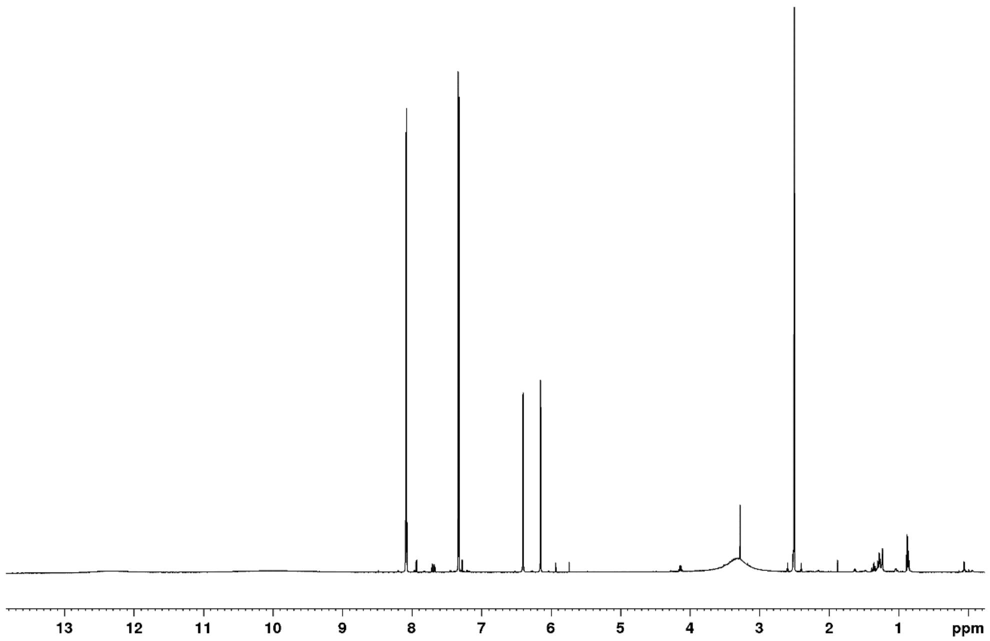

Figure S14. ^{1}H NMR Spectrum of compound **14** (700.13 MHz for ^{1}H, DMSO-d_6).

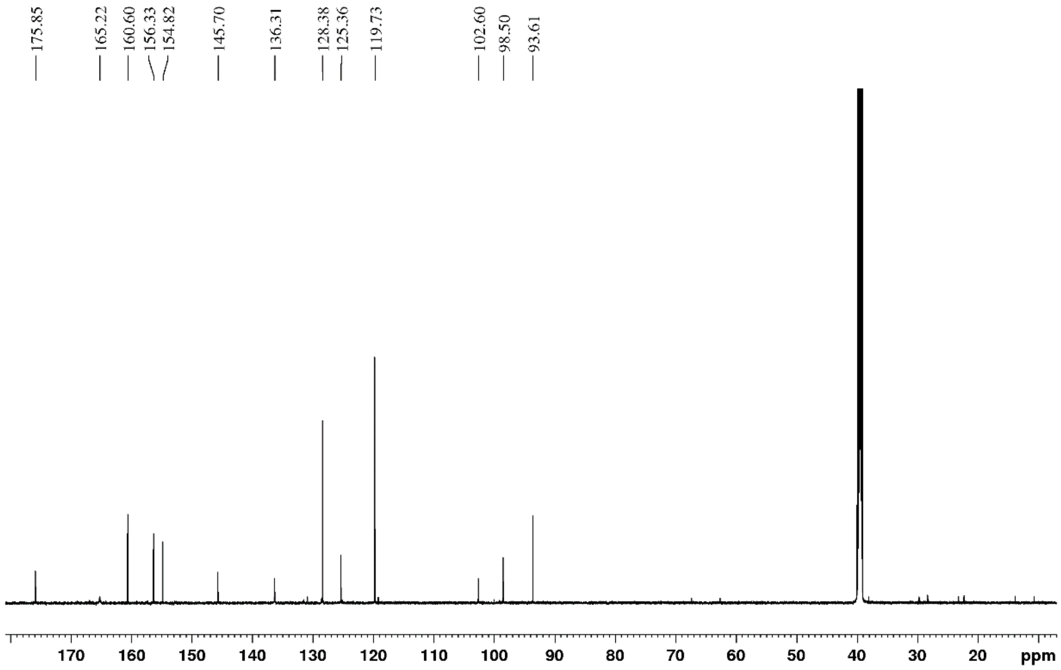

Figure S15. ^{13}C NMR Spectrum of compound **14** (176.05 MHz for ^{13}C, DMSO-d_6).

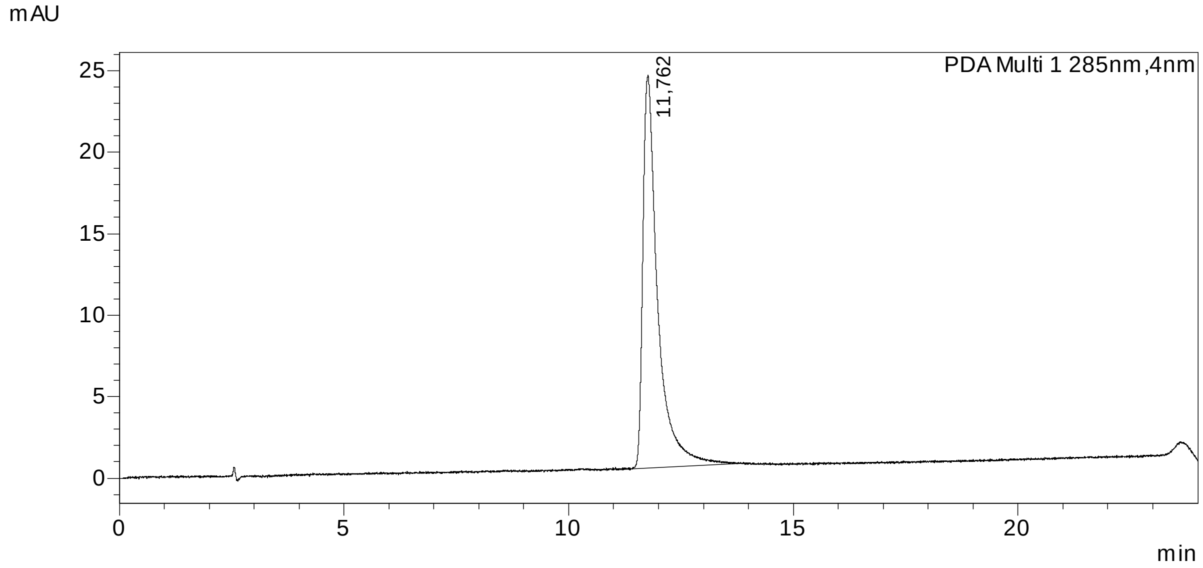

Figure S16. HPLC chromatogram of product **14** detected at the retention time of 11.76 min (purity 99%).

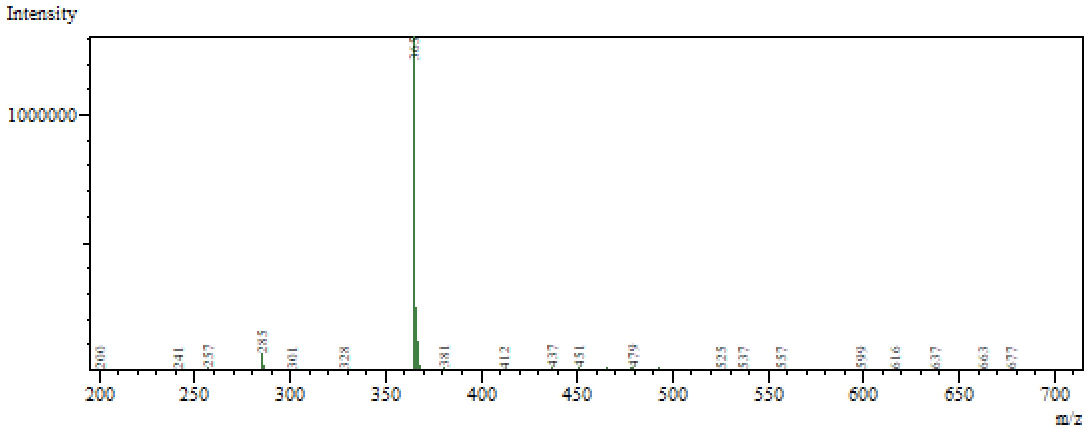

Figure S17. ESI-MS Spectrum of product **14** in negative mode: calculated *m/z* for $[M-H]^-$ is 365.29, found 365.

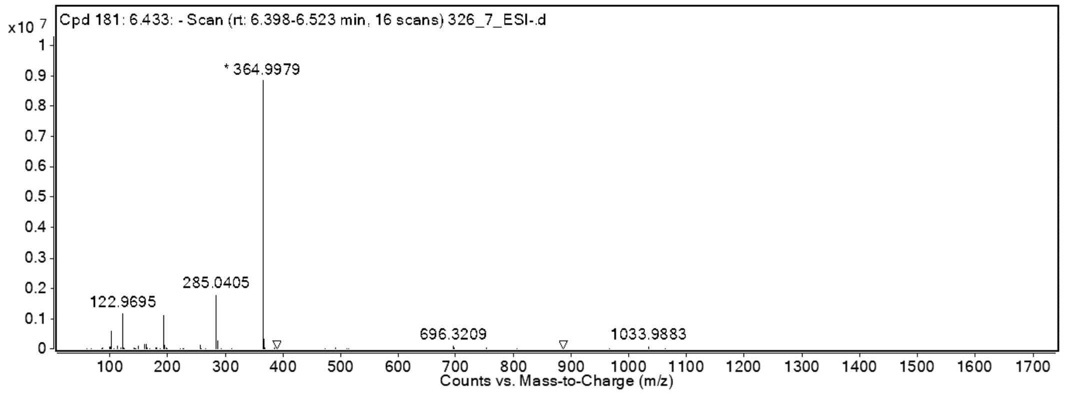

Figure S18 HRMS spectrum of product **14** in negative ion mode ($[M-H]^-$, *m/z* 364.9979). Calculated for $C_{15}H_{10}O_9S$ = 364.99728, found 364.9979 (1.6986 ppm). Compound **14** fragmentation $[M-SO_3]^-$: calculated 285.04046 *m/z*, found 285.0405 *m/z* (0.1403 ppm).

Table S5. ^{1}H and ^{13}C NMR data for the compound kaempferol-7-*O*-sulfate (**15**) in a sample containing compound **14.** The molar ratio of compounds **15/14** in the mixture was 3/7; 700.13 MHz for ^{1}H, 176.05 MHz for ^{13}C, DMSO-d_6, 30 °C.

Atom	δ_C	m.	δ_H	n_H	m.	*J* [Hz]
**2**	147.51	s	-	0		
**3**	135.92	s	-	0		
**4**	176.09	s	-	0		
**5**	159.67	s	-	0		
**6**	101.24	d	6.549	1	d	2.0
**7**	159.20	s	-	0		
**8**	97.42	d	6.981	1	d	2.0
**9**	155.09	s	-	0		
**10**	104.93	s	-	0		
**1'**	121.48	s	-	0		
**2'**	129.55^{x}	d	8.081	2	m	Σ*J* = 9.0
**3'**	115.41^{x}	d	6.934	2	m	Σ*J* = 9.0
**4'**	159.28	s	-	0		

^{x} ... 2C

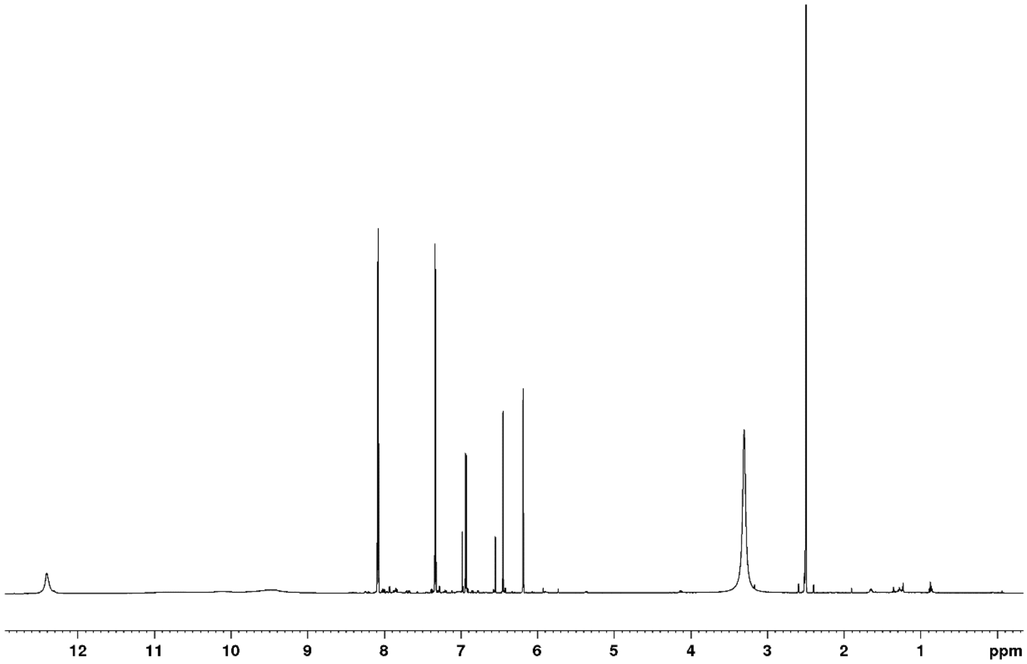

Figure S19. ^{1}H NMR Spectrum of compound **15** in a mixture with **14** (700.13 MHz for ^{1}H, DMSO-d_6).

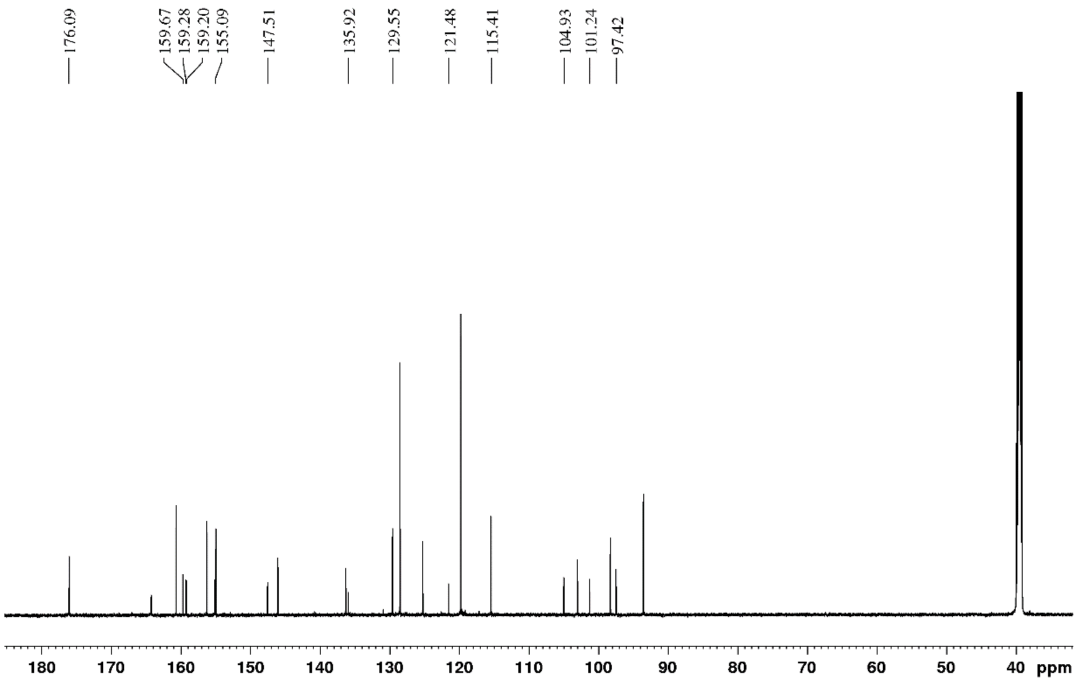

Figure S20. ^{13}C NMR Spectrum of compound **15** in the mixture with **14** (its peaks are not labeled for the sake of clarity; 176.05 MHz for ^{13}C, DMSO-d_6).

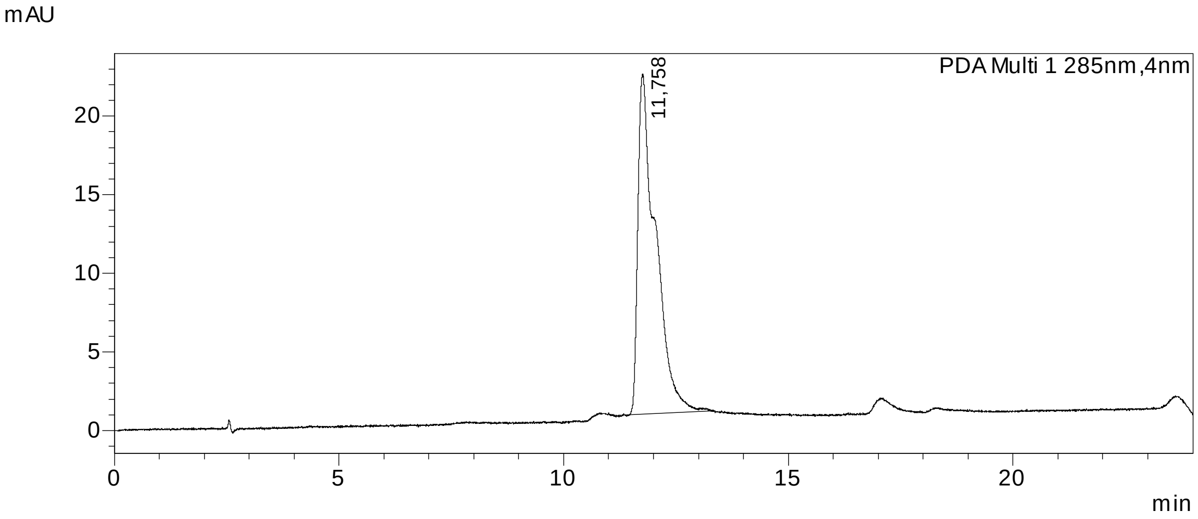

Figure S21. HPLC chromatogram of products **14** and **15** detected at retention time 11.76 min (purity 92%).

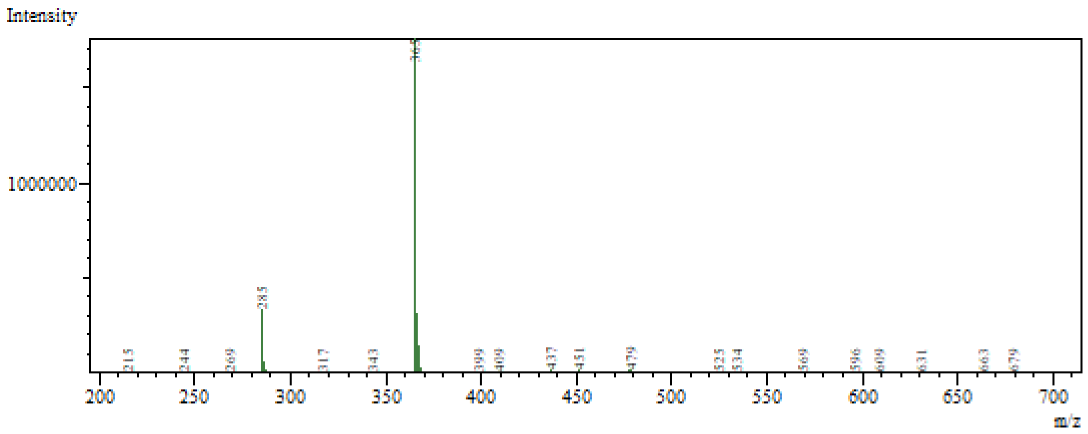

Figure S22. ESI-MS Spectrum of product **15** at in the negative mode: calculated *m/z* for $[M\text{-}H]^-$ is 365.29, found 365.

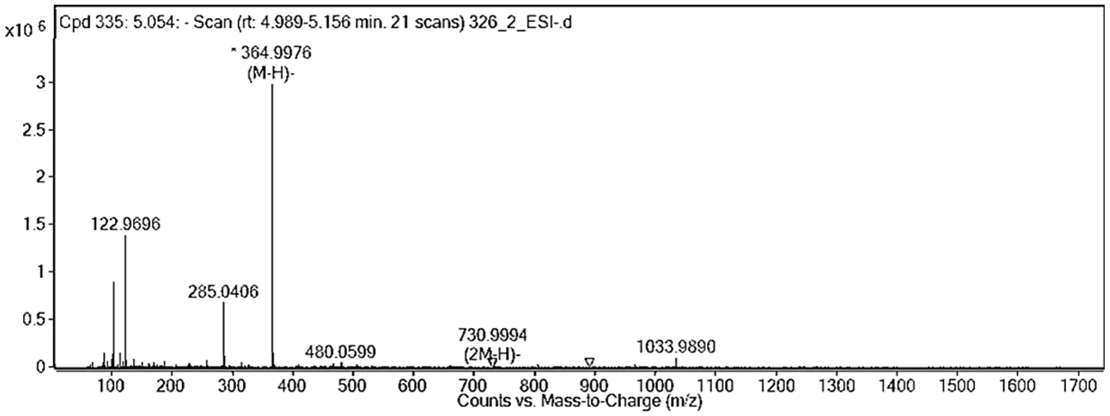

Figure S23. HRMS spectrum of product **15** in negative ion mode ($[M\text{-}H]^-$, *m/z* 364.9979). Calculated for $C_{15}H_{10}O_9S$ = 364.99728, found 364.9979 (1.6986 ppm). Compound **15** fragmentation $[M\text{-}HSO_3]^-$: calculated 285.04046 *m/z*, found 285.0405 *m/z* (0.1403 ppm). Also found dimer [2M-H] -, *m/z* 730.9994, calculated 731.00183.

Table S6. ^{1}H and ^{13}C NMR data for the compound kaempferol-7,4'-*O*-disulfate (**16**); 700.13 MHz for ^{1}H, 176.05 MHz for ^{13}C, DMSO-d_6, 30 °C.

Atom	δ_C	m.	δ_H	n_H	m.	*J* [Hz]
**2**	146.83	s	-	0		
**3**	136.75	s	-	0		
**4**	176.41	s	-	0		
**5**	159.70	s	-	0		
**6**	101.29	d	6.576	1	d	2.0
**7**	159.33	s	-	0		
**8**	97.52	d	6.986	1	d	2.0
**9**	155.22	s	-	0		
**10**	105.04	s	-	0		
**1'**	125.14	s	-	0		
**2'**	128.61^x	d	8.130	2	m	Σ*J* = 9.0
**3'**	119.74^x	d	7.339	2	m	Σ*J* = 9.0
**4'**	155.04	s	-	0		

^{x} … 2C

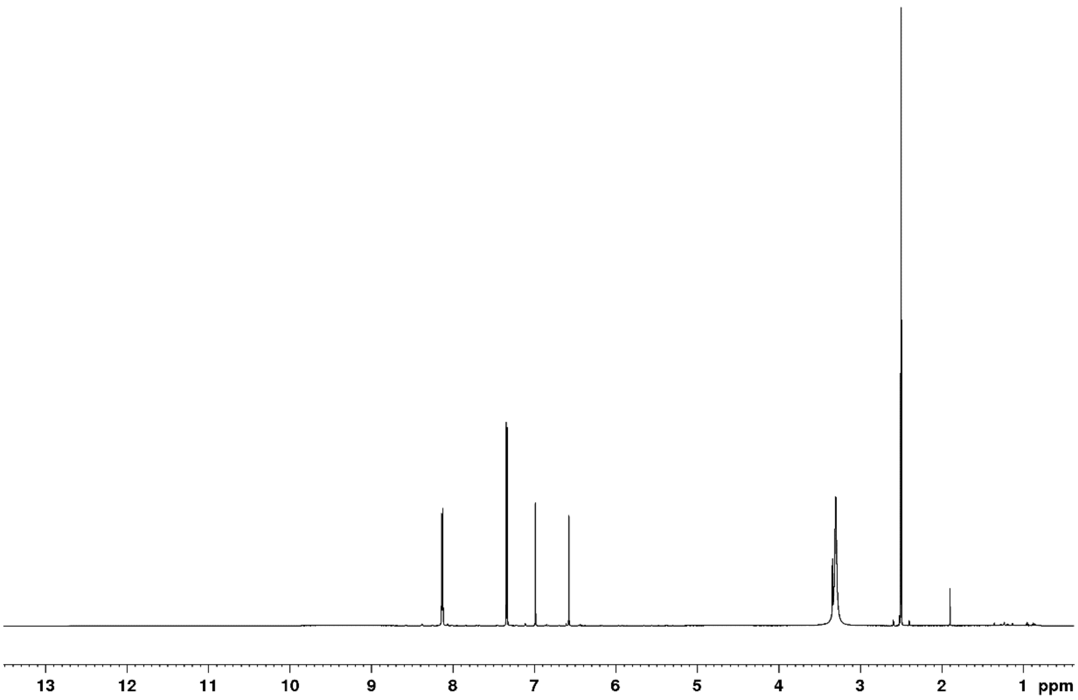

Figure S24. ^{1}H NMR Spectrum of compound **16** (700.13 MHz for ^{1}H, DMSO-d_6).

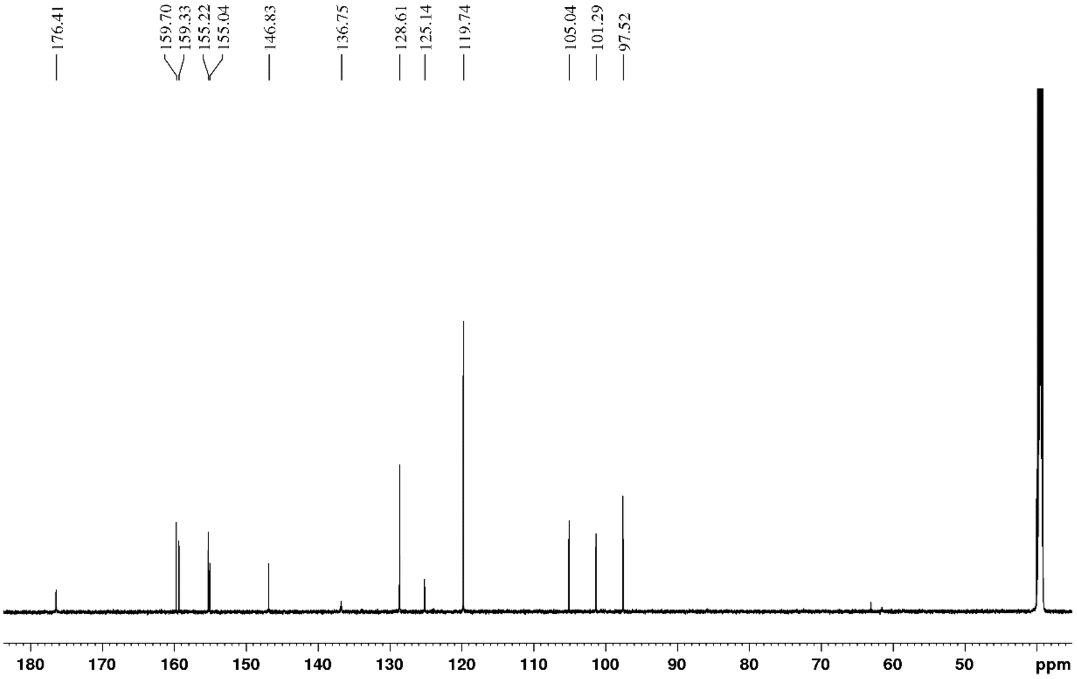

Figure S25. ^{13}C NMR Spectrum of compound **16** (176.05 MHz for ^{13}C, DMSO-d_6).

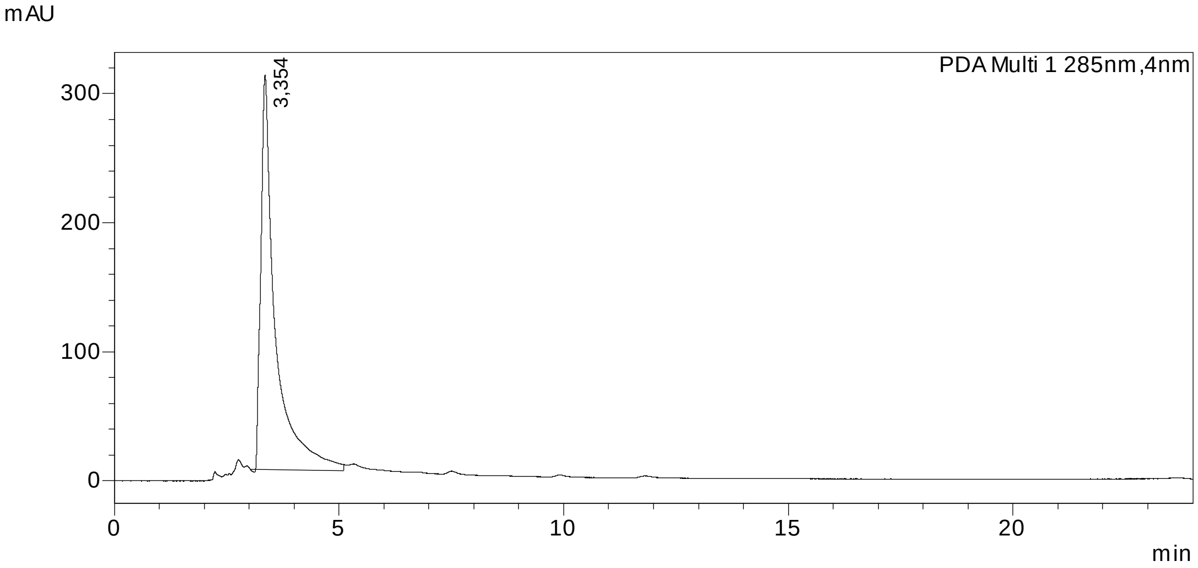

Figure S26. HPLC chromatogram of compound **16** detected at retention time 3.3 min (purity 98%)

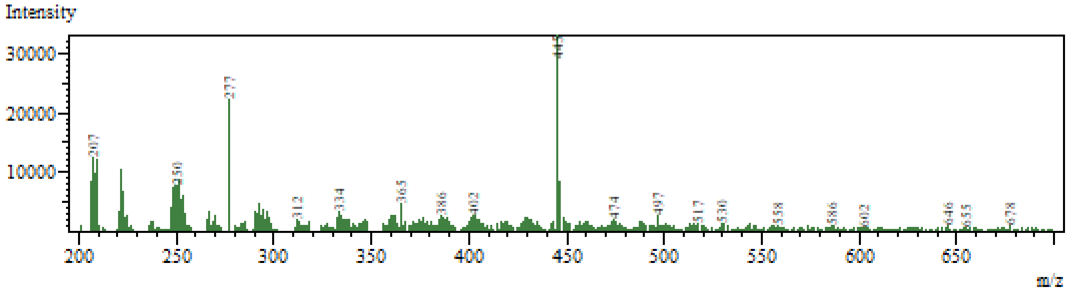

Figure S27. ESI-MS Spectrum of compound **16** in negative mode: calculated *m/z* for $[M\text{-}H]^-$ 445.34, found 445.

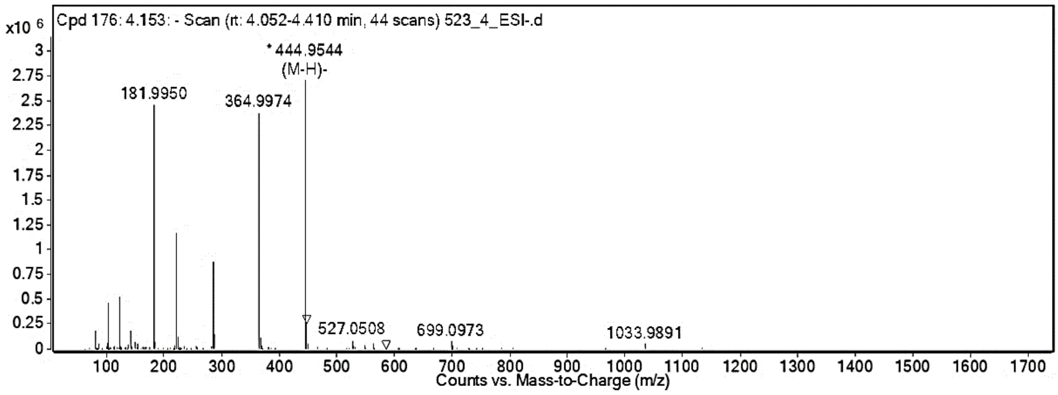

Figure S28. HRMS spectrum of product **16** in negative ion mode ($[M\text{-}H]^-$, *m/z* 444.9544). Calculated for $C_{15}H_{10}O_{12}S_2$ = 444.95409, found 444.9544 (0.6967 ppm). Monosulfate fragment of compound **16** $[M\text{-}SO_3\text{-}H]^-$ was found at *m/z* 364.9974, calculated 364.99728 (0.3288 ppm). Double charge molecule state $[M\text{-}2H]^{-2}$, *m/z* 221.9737, and monosulfate double charge molecule state $[M\text{-}SO_3\text{-}2H]^{-2}$, 181.9950, were found.

Table S7. ^{1}H and ^{13}C NMR data for the compound kaempferol-3,4'-*O*-disulfate (**17**); 700.13 MHz for ^{1}H, 176.05 MHz for ^{13}C, DMSO-d_6, 30 °C.

**Atom**	δ_C	**m.**	δ_H	**n$_H$**	m.	***J* [Hz]**
**2**	155.69	s	-	0		
**3**	132.82	s	-	0		
**4**	177.64	s	-	0		
**5**	161.23	s	-	0		
**6**	98.61	d	6.153	1	d	2.1
**7**	164.70	s	-	0		
**8**	93.59	d	6.405	1	d	2.1
**9**	156.19	s	-	0		
**10**	103.91	s	-	0		
**1'**	124.85	s	-	0		
**2'**	129.66^x	d	8.122	2	m	Σ*J* = 9.0
**3'**	119.08^x	d	7.252	2	m	Σ*J* = 9.0
**4'**	155.53	s	-	0		

^{x} … 2C

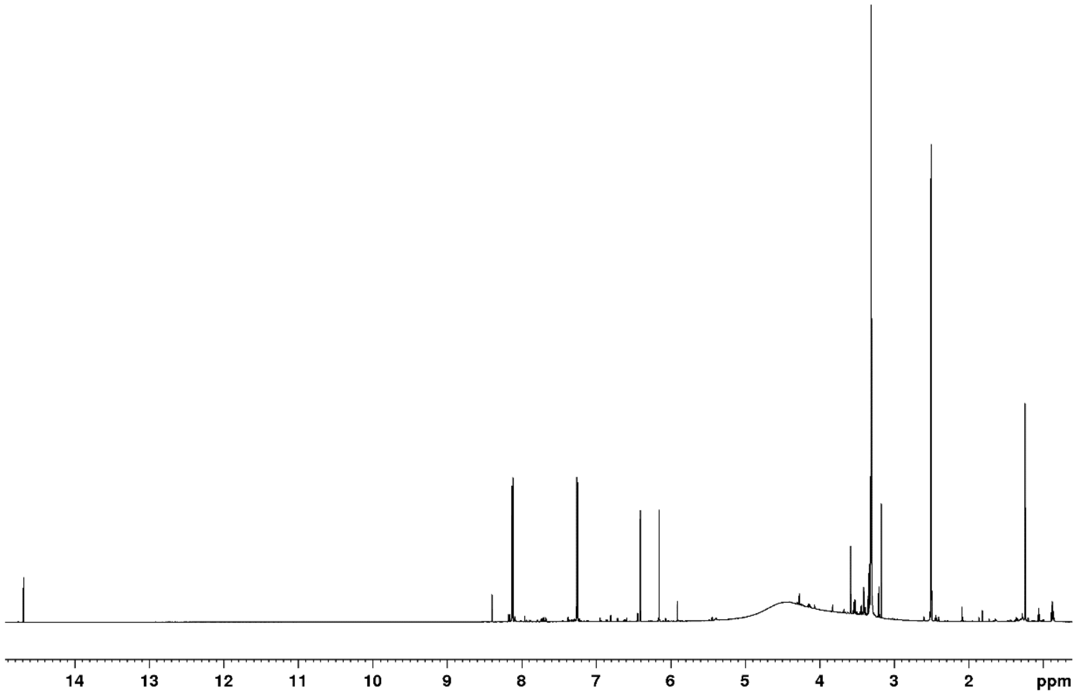

Figure S29. ^{1}H NMR Spectrum of compound **17** (700.13 MHz for ^{1}H, DMSO-d_6).

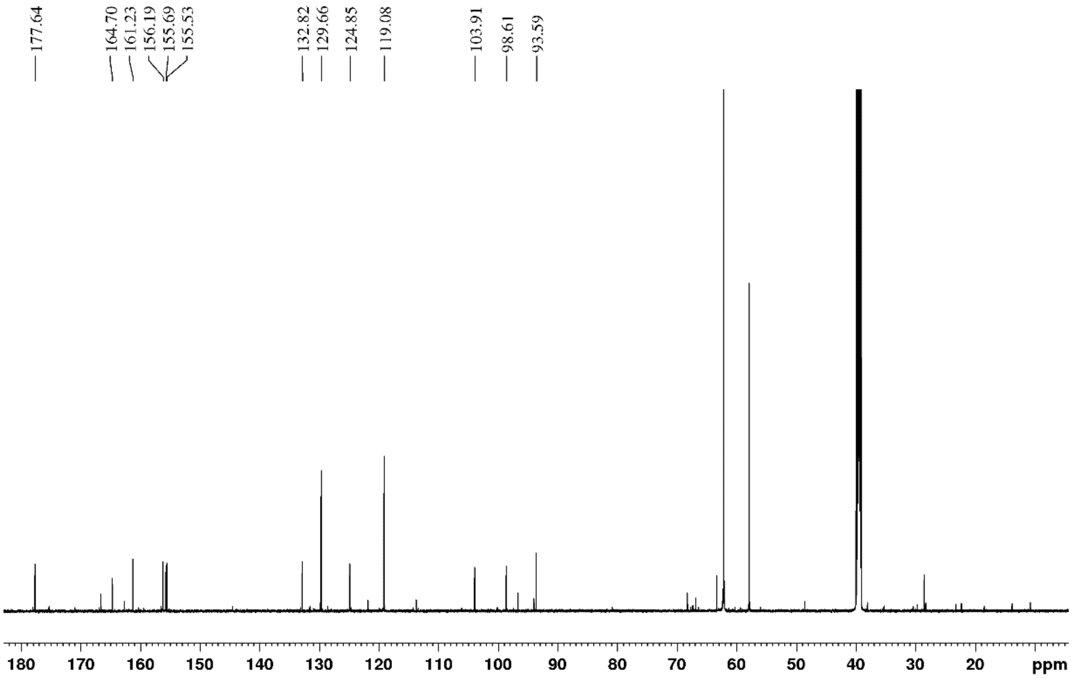

Figure S30. ^{13}C NMR Spectrum of compound **17** (176.05 MHz for ^{13}C, DMSO-d_6).

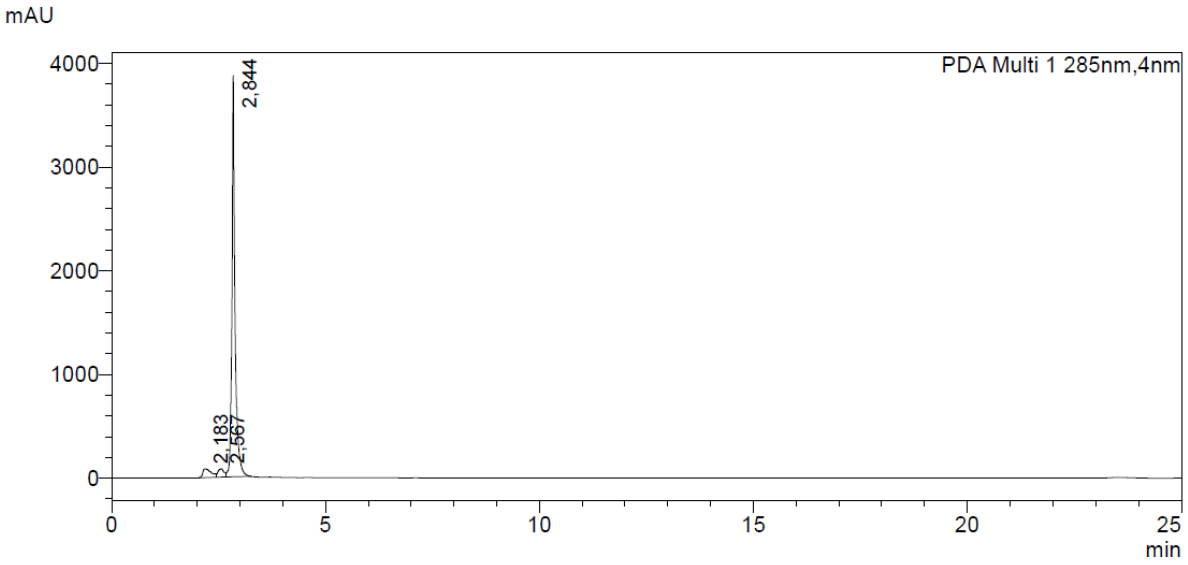

Figure S31. HPLC chromatogram of compound **17** detected at 2.8 min (purity 96%) .

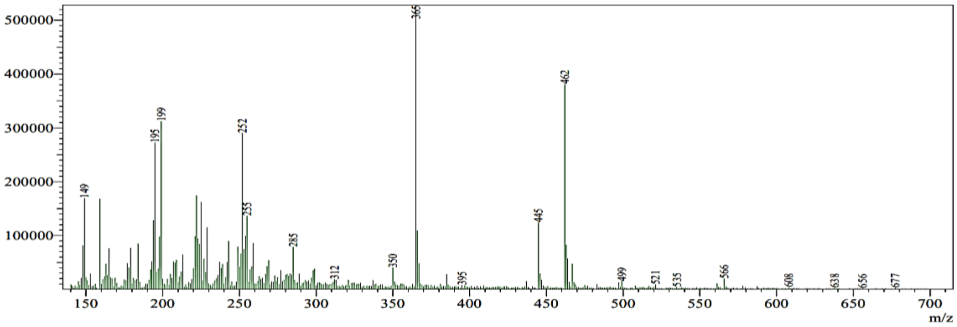

Figure S32. ESI-MS Spectrum of compound **17** at 2.8 min in negative mode: calculated *m/z* for $[M-H]^-$ is 445.34, found445. The product partially degraded during ionization and *m/z* for monosulfate was found: $[M-H]^-$ = 365, calculated 365.29.

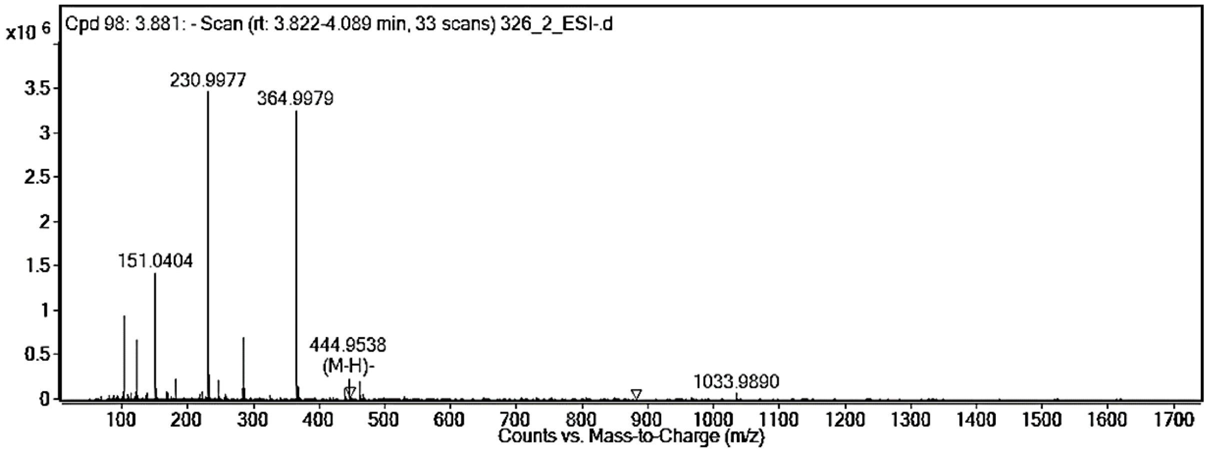

Figure S33. HRMS spectrum of product **17** in negative ion mode ($[M-H]^-$, *m/z* 444.9538). Calculated for $C_{15}H_{10}O_{12}S_2$ = 444.95409, found 444.9538 (-0.6517 ppm). Monosulfate fragment of compound **17** $[M-SO_3-H]^-$ was found at *m/z* 364.9979, calculated 364.99728 (1.4247ppm). Double charge molecule state $[M-2H]^{-2}$, *m/z* 221.9737 was found. Fragments: calculated for $C_8H_7O_6S$ = 230.996882 *m/z*, observed 230.9977; calculated for $C_8H_7O_3$= 151.040068 *m/z*, observed 151.0404.

Table S8. The comparison of ^{13}C NMR data of products **14**-**17** with the parent compound kaempferol (**8**)[a]

Atom	kaempferol (8)	4'-*O*-sulfate (14)	7-*O*-sulfate (15)	7,4'-*O*-disulfate (16)	3,4'-*O*-disulfate (17)
**2**	146.74	145.70	147.51	146.83	**155.69**
**3**	135.56	136.31	135.92	136.75	**132.82**
**4**	175.82	175.85	176.09	176.41	**177.64**
**5**	160.63	160.60	159.67	159.70	161.23
**6**	98.11	98.50	**101.24**	**101.29**	98.61
**7**	163.81	165.22	**159.20**	**159.33**	164.70
**8**	93.38	93.61	**97.42**	**97.52**	93.59
**9**	156.10	156.33	155.09	155.22	156.19
**10**	102.96	102.60	**104.93**	**105.04**	103.91
**1'**	121.59	**125.36**	121.48	**125.14**	**124.85**
**2'**	129.41	128.38	129.55	128.61	129.66
**3'**	115.35	**119.73**	115.41	**119.74**	**119.08**
**4'**	159.10	**154.82**	159.28	**155.04**	**155.53**

[a] significant changes induced by sulfation are highlighted in **bold**
